# Mulberry leaf alleviates streptozotocin-induced diabetic rats by attenuating NEFA signaling and modulating intestinal microflora

**DOI:** 10.1038/s41598-017-12245-2

**Published:** 2017-09-21

**Authors:** Yao Sheng, Shujuan Zheng, Tianshi Ma, Chuanhai Zhang, Xiaoqun Ou, Xiaoyun He, Wentao Xu, Kunlun Huang

**Affiliations:** 1 Beijing Advanced Innovation Center for Food Nutrition and Human Health, College of Food Science and Nutritional Engineering, China Agricultural University, Beijing, 100083 China; 2 Key Laboratory of Safety Assessment of Genetically Modified Organism (Food Safety), Ministry of Agriculture, Beijing, 100083 China

## Abstract

Improvement of hyperglycemia through dietotherapy/herbal remedy is an effective approach to treating diabetes. In this study, mulberry leaf, famous for silkworm’s special food and therapeutic value without any side effects, alleviated diabetes by attenuating NEFA signaling and modulating intestinal microflora. Mulberry leaf treatment significantly reduce fasting blood-glucose and HbA1c, ameliorate the blood lipid profile and improve insulin resistance in streptozotocin-induced diabetic rats. Mechanistically, we found that mulberry leaf inhibited NEFA signaling by reducing downstream signaling in the NEFA pathway, further verified by reduced PKC and improved cellular energy homeostasis based on restored expression of PGC-1α, AK2, OXPHOS and adiponectin. Mulberry leaf treatment also restored the phyla *Bacteroidetes* and *Proteobacteria* and class *Clostridia*, which were associated with insulin resistance and diabetes. Our findings reveal that mulberry leaf is an edible with therapeutic potential for diabetes and may provide a novel dietotherapy/herbal remedy to the treatment of diabetes.

## Introduction

The increased prevalence of diabetes has focused attention on a worldwide issue that significantly affects human health. The diabetic population reached 415 million in 2015 and is predicted to increase to 642 million in 2040 worldwide according to the International Diabetes Federation (IDF)^1^. Hyperglycemia is the main symptom of diabetes which also leading to many complications such as, heart disease, stroke, chronic kidney failure^2–4^. Conventional therapy such as insulin and some oral medications can cause hypoglycemia^5^ and bring economic burden to patients. Therefore, some more acceptable strategy is urgently needed to develop. Recently, dietotherapy/herbal remedy posed potential in treating patients with diabetes for its advantage such as, safety, low cost and effectiveness^6,7^.

Mulberry leaves have been used as traditional medicine to prevent and cure diabetes^8^. Several pharmacologically important compounds, such as polyphenols, flavonoids, terpenoids and polysaccharides, are frequently reported to play roles in its anti-hyperglycemic effect^9^. In particular, 1-deoxynojirimycin (DNJ), which is abundant in mulberry leaf (*Morus alba L*.), is believed to be a typical naturally occurring imino sugar with potent biological activity^10^. However, the underlying anti-hyperglycemic mechanism of mulberry leaves has not been determined completely.

Diabetes is associated with insulin resistance and/or pancreatic islet β-cell dysfunction. The release of non-esterified fatty acids (NEFAs) may be the single most important factor in modulating insulin sensitivity. Increased NEFA levels are observed in obesity and type II diabetes, and they are associated with the insulin resistance observed in both^11,12^. Insulin resistance develops within hours of an acute increase in plasma NEFA levels in humans^13^. Conversely, insulin-mediated glucose uptake and glucose tolerance improve with an acute decrease in the NEFAs levels after treatment with an antilipolytic agent, acipimox^14^. In addition, NEFAs are important for normal β-cell function and they potentiate insulin release in response to glucose and non-glucose secretagogues^15,16^. This might involve a mechanism that depends on protein kinase c (PKC). NEFAs promote the generation of fatty acyl-CoA, which increases insulin release both by directly stimulating secretory granule exocytosis and by PKC activation^16^. NEFAs induce insulin resistance and impair β-cell function, making them likely culprits.

Recently, accumulated evidence has suggested that the intestinal microbiota plays an important role in the pathogenesis of diabetes^17,18^. Many articles have reported a correlation between changes in the intestinal microbiota and markers of diabetes. *Lactobacillus* species positively correlate with fasting glucose and glycosylated hemoglobin (HbA1c) levels, whereas *Clostridium* species negatively correlate with fasting glucose, HbA1c and insulin levels^19^. A recent study suggests that a higher blood glucose concentration may be predicted by a reduction in the proportion of anaerobes, particularly *Bacteroides* ^20^. The intestinal microbiota may influence the host by affecting the body weight, bile acid metabolism, proinflammatory activity^21^, insulin resistance^22^, and modulation of gut hormones^23^. Therefore, therapy that modulates the intestinal microbiota may benefit glucose metabolism and improve insulin resistance in the host.

In our study, the anti-hyperglycemic effect of mulberry leaves which mixed into the food was studied with streptozotocin (STZ)-induced diabetes rats, and the potential mechanisms were explored. Our research also reveal that mulberry leaf mixed in food plays a well enough role in combating diabetes, which may provide a novel therapeutic approach to the treatment of hyperglycemia.

## Results

### Mulberry leaf was rich in bioactive substances

Bioactive substances of mulberry leaf, such as polysaccharide, DNJ, polyphenols and flavonoid, are shown in Table 1. The concentration of polysaccharide was 134.68 mg/g mulberry leaf powder. The level of DNJ reached 1.281 mg/g mulberry leaf powder, which was slightly higher than that given in other reports^24,25^. In addition, mulberry leaf was rich in rutin (29.86 mg/g), gallic acid (16.94 mg/g), chlorogenic acid (6.89 mg/g), and GABA (14.62 mg/g). Nutritional proximates, vitamins, minerals and fatty acids of mulberry leaf are shown in Supplementary Materials Tables S3 and S4.

**Table 1:** The concentration of bioactive substances of mulberry leaf.

Substance	Concentration
Polysaccharide (mg/g)	134.68 ± 5.34
1-DNJ (mg/g)	1.281 ± 0.241
Rutin (mg/g)	29.86 ± 1.03
Chlorogenic acid (mg/g)	6.89 ± 0.386
Gallic acid	16.94 ± 0.815
β-sitosterol (mg/g)	0.717 ± 0.064
γ- aminobutyric acid (mg/g)	14.62 ± 1.081
Carotene (mg/g)	0.057 ± 0.006

Value = mean ± SD (N = 3).

### Mulberry leaf reduced food and caloric intake

**Figure 1:**
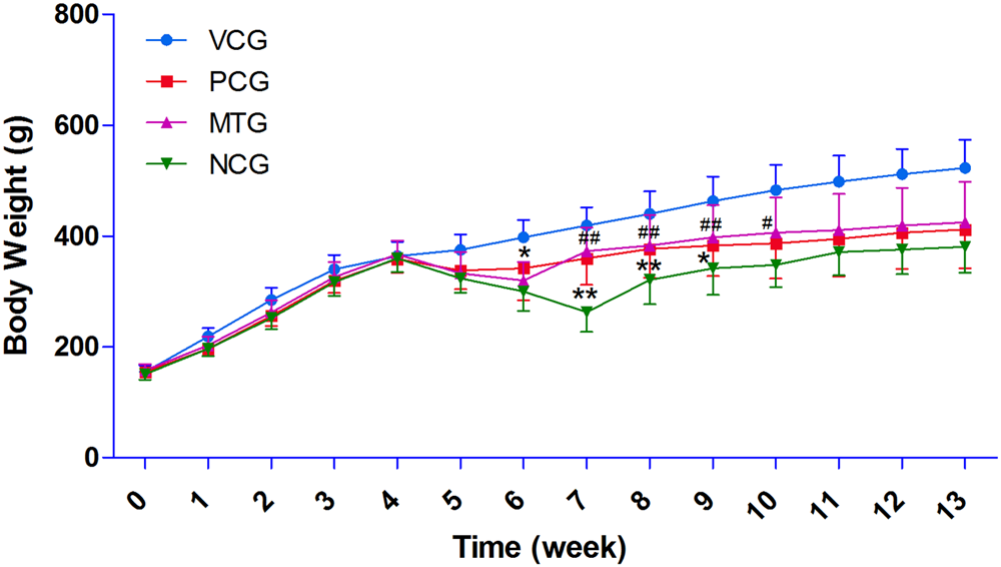
Body weight of rats during experimental time. Acute weight loss was observed in all groups treated with STZ. Both mulberry leaf and glibenclamide could protect the animals from acute weight loss (6–10^th^ weeks) to a certain extent. During the 7–8^th^ weeks, the body weights of rats in both PCG and MTG were significantly (P < 0.01) higher than NCG. *P < 0.05 PCG compared with NCG; **P < 0.01 PCG compared with NCG; ^#^P < 0.05 MTG compared with NCG; ^##^P < 0.01 MTG compared with NCG. Value = mean ± SD (N = 6).

STZ treatment significantly reduced the body weights (Fig. 1 and Table S5) of rats. Both mulberry leaf and glibenclamide could somewhat protect the animals from acute weight loss (6–10^th^ weeks). During the 7–8^th^ weeks, the body weights of rats in both PCG and MTG were significantly (P < 0.01) higher than NCG. In addition, STZ-induced diabetic rats had higher food intake and total caloric intake (Table 2); however, they had less weight gain than did the other groups. The results indicated that mulberry leaf treatment could improve the food efficiency ratio (weight gain/food intake) through reducing the food and caloric intake and decreasing the body weight of diabetic rats.

**Table 2:** Food and caloric intakes of the rats in each cage.

Weeks	1	2	3	4	5	6	7	8	9	10	11	12	13	food efficiency ratio	total caloric intake (kcal)
VCG	760 ± 38	890 ± 8	960 ± 20	741 ± 2	946 ± 10	962 ± 4	978 ± 1	1039 ± 35	824 ± 57	885 ± 20	895 ± 1	770 ± 39	754 ± 35	12.91%	9750
PCG	598 ± 17	697 ± 51	740 ± 75	666 ± 1	706 ± 33	761 ± 31	817 ± 30	993 ± 23	1013 ± 30	934 ± 46	899 ± 3	680 ± 36	833 ± 47	9.96%	12214
MTG	602 ± 35	712 ± 35	807 ± 69	651 ± 5	684 ± 17	793 ± 1	882 ± 47	850 ± 38	867 ± 164	791 ± 51	948 ± 77	799 ± 100	816 ± 16	10.46%	12054
NCG	556 ± 3	620 ± 28	735 ± 6	654 ± 39	737 ± 26	880 ± 23	1024 ± 21	1285 ± 75	830 ± 186	1239 ± 64	1231 ± 36	1010 ± 39	829 ± 99	7.93%	13741

Value = mean ± SD (N = 2).

### Anti-hyperglycemic effect of mulberry leaf

**Figure 2:**
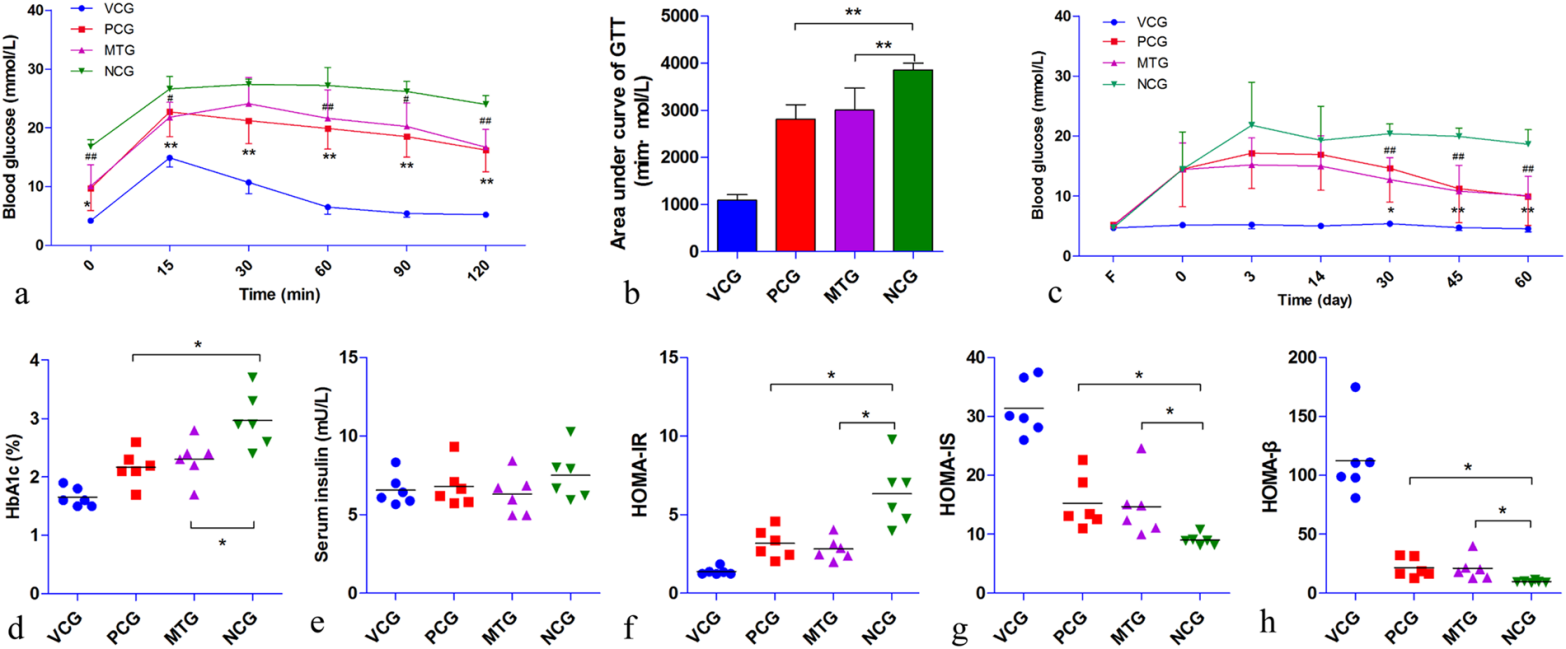
Antihyperglycemic effect of mulberry leaf. (**a**) GTT, both mulberry leaf and glibenclamide could enhance insulin sensitivity of diabetic rat, *P < 0.05 PCG compared with NCG, **P < 0.01 PCG compared with NCG, ^#^P < 0.05 MTG compared with NCG, ^##^P < 0.01 MTG compared with NCG. (**b**) Area under curve of GTT, **P < 0.01 compared with NCG. (**c**) FBG levels of rats, FBG of NCG maintained high level throughout the trial, however, both mulberry leaf and glibenclamide could significantly reduce FBG of diabetic rat, *P < 0.05 PCG compared with NCG, **P < 0.01 PCG compared with NCG, ^##^P < 0.01 MTG compared with NCG. (**d**) HbA1c levels of rats, both mulberry leaf and glibenclamide treatments significantly inhibited glycation procedure of diabetic rat, *P < 0.05 compared with NCG. (**e**) Serum fasting insulin level of rats. (**f**) HOMA-IR, both mulberry leaf and glibenclamide decreased insulin resistance, *P < 0.05 compared with NCG. (**g**) HOMA-IS, both mulberry leaf and glibenclamide increased insulin sensitivity, *P < 0.05 compared with NCG. (**h**) HOMA-β, both mulberry leaf and glibenclamide improved pancreas islet β-cell function, *P < 0.05 compared with NCG. Value = mean ± SD (N = 6).

STZ treatment successfully induced hyperglycemia in rats (Fig. 2c). The fasting blood-glucose (FBG) of NCG maintained a high level throughout the trial. Both mulberry leaf and glibenclamide could significantly reduce the FBG of diabetic rats (p < 0.05) after 30 days of treatment (Fig. 2c and Table S7). The GTT result indicted that both mulberry leaf and glibenclamide could enhance the insulin sensitivity of diabetic rats (Fig. 2a and Table S6). The areas under curve of GTT in both PCG and MTG were significantly (P < 0.01) lower than that of NCG (Fig. 2b). The HbA1c level of NCG increased dramatically compared to VCG as persistent hyperglycemia (Fig. 2d and Table S8). What’s more, mulberry leaf treatment significantly inhibited the glycation procedure of diabetic rats as the same therapeutic effect as glibenclamide (p < 0.05). Although the fasting serum insulin (FSI) level was unchanged between each group (Fig. 2e and Table S9), the homeostasis model assessment (HOMA, Fig. 2f,g and Tables 10–12 howed significantly reduced HOMA-IR and increased HOMA-IS and HOMA-β levels in both MTG and PCG compared with NCG. In addition, pathological data indicated that mulberry leaf could ameliorate pancreas islet damage by STZ (Fig. S1). In general, mulberry leaf could significantly reduce the FBG and HbA1c levels and improve the insulin sensitivity of diabetic rats.

### Anti-hyperlipidemic effect of mulberry leaf

**Figure 3:**
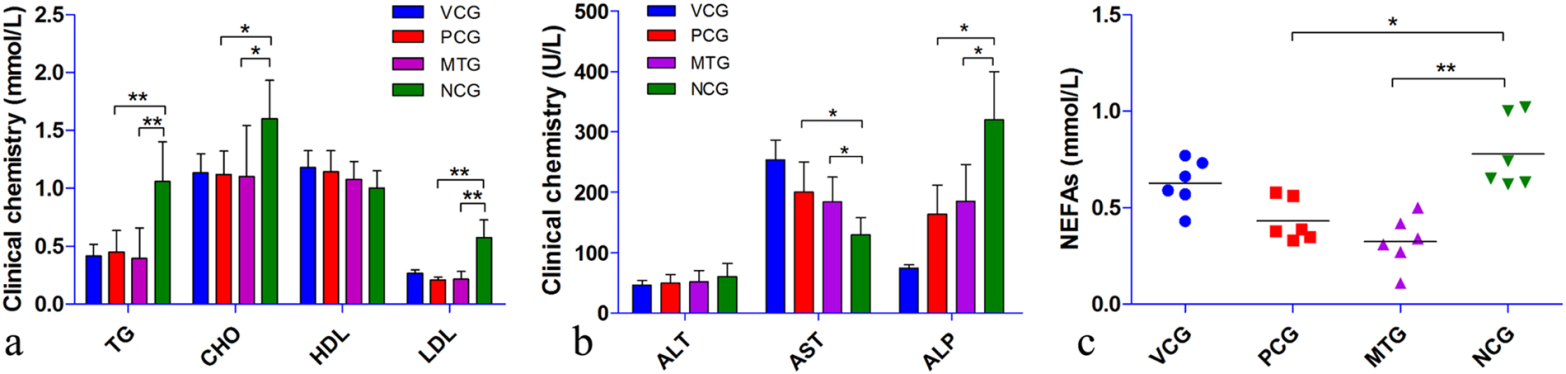
Anti-hyperlipidemic effect of mulberry leaf. (**a**) Blood lipid profile, the levels of TG, CHO and LDL were reduced with mulberry leaf treatment. (**b**) Enzymatic activity, AST level was reduced in NCG and restored by mulberry leaf treatment, ALP level increased in NCG and was significantly reduced with mulberry leaf treatment. (**c**) The level of serum NEFAs, the level of serum NEFAs was significantly elevated in diabetic rats, however, could be significantly reduced with mulberry leaf treatment. *P < 0.05 compared with NCG, **P < 0.01 compared with NCG. Value = mean ± SD (N = 6).

Diabetic rats of NCG had an aggravated blood lipid profile (elevated TG, CHO and LDL levels and slightly reduced HDL level) (Fig. 3a). Mulberry leaf treatment significantly reduced both the TG and LDL levels (P < 0.01) compared with NCG. In addition, the CHO level of MTG was also significantly (P < 0.05) decreased. While, no statistically significant differences were observed in the serum HDL and ALT levels between each group (Fig. 3b). Interestingly, the AST level was reduced in NCG. However, ALP increased in NCG and was significantly reduced (P < 0.05) with mulberry leaf and glibenclamide treatments. The results indicated that mulberry leaf could significantly ameliorate the blood lipid profile and hyperglycemia.

### Inhibition of NEFA signaling pathway

**Figure 4:**
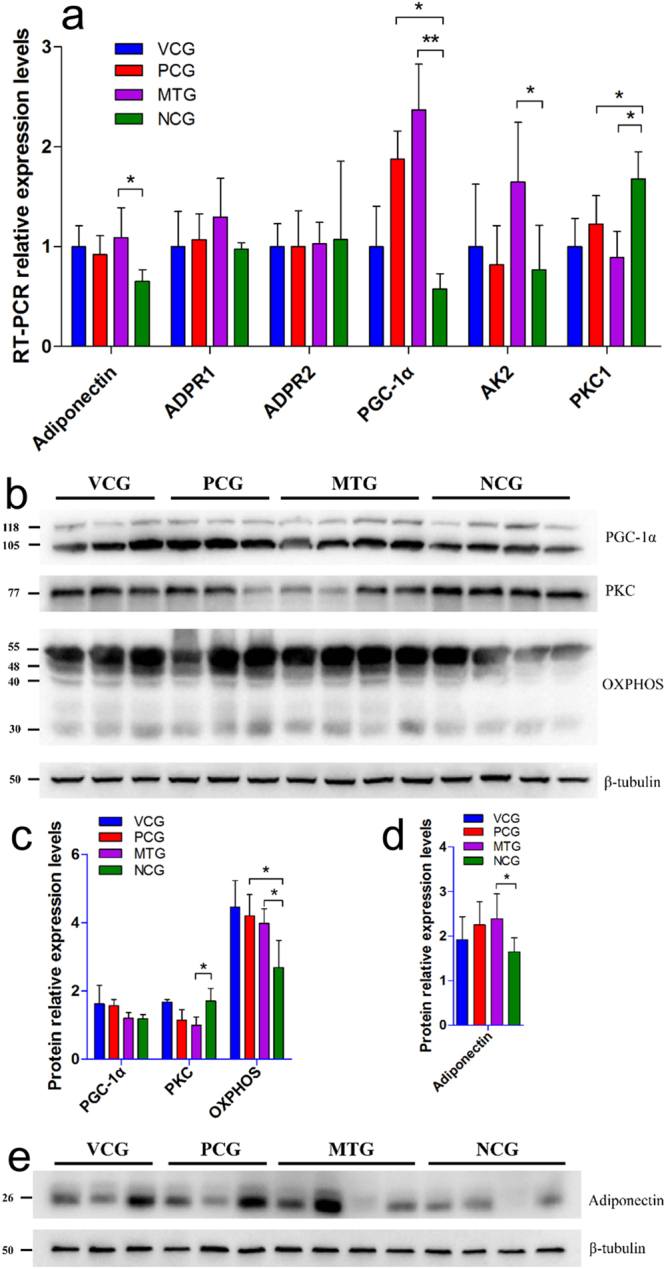
The results of RT-PCR and western blot. (**a**) RT-PCR, mulberry leaf decreased PKC and increased PGC-1α and AK2 in liver tissue, and increased adiponectin expression in subcutaneous fat. *P < 0.05 compared with NCG, **P < 0.01 compared with NCG. Value = mean ± SD (N = 6). (**b,c**) Western blot for protein in liver tissue, mulberry leaf decreased PKC and increased OXPHOS expression. β-tubulin serves as a loading control. For presentation purposes additional lanes were excised. PKC, PGC-1α, OXPHOS and β-tubulin were cropped from different parts of the same gel. These proteins were stained one by one after using Restore™ Western Blot Stripping Buffer (Thermo Scientific, USA). Full-length gels for each protein were included in Supplementary Information Fig. S2. (**d,e**) Western blot for protein in subcutaneous fat, mulberry leaf increased adiponectin expression in subcutaneous fat. β-tubulin serves as a loading control. For presentation purposes additional lanes were excised. Adiponectin and β-tubulin were cropped from different parts of the same gel. Full-length gels both proteins were included in Supplementary Information Fig. S3. *P < 0.05 compared with NCG, **P < 0.01 compared with NCG. Value = mean ± SD (N = 3–4).

As an important factor in modulating insulin sensitivity, NEFAs were elevated in diabetic rats (Fig. 3c) compared with vehicle control. The increased circulating NEFAs can induce insulin resistance and impair β-cell function via PKC activation^16^. Diabetic rats of NCG had a significantly elevated level of PKC, as indicated by the results of RT-PCR (Fig. 4a) and western blot (Fig. 4b and c). However, mulberry leaf could significantly reduce NEFAs (P < 0.01) and PKC (P < 0.05) levels.

Enhanced NEFA signaling affect energy homeostasis^26^: circulating NEFAs reduce adipocyte and muscle glucose uptake, and also promote hepatic glucose output^13,27^; in addition, NEFAs decrease glucose sensing by reducing mitochondrial oxidative phosphorylation (OXPHOS), ATP synthesis^28^. Decreased peroxisome proliferator activated receptor gamma coactivator 1-alpha (PGC-1α) and adenylate kinase 2 (AK2) transcription levels and reduced OXPHOS proteins and PGC-1α at the post-translation level were observed in diabetic rats without any treatment. However, mulberry leaf could significantly restore the expression of OXPHOS and PGC-1α at the end of the trial. Significantly increased PGC-1α (P < 0.01) and AK2 (P < 0.05) transcription levels were also observed in MTG compared with NCG (Fig. 4b and c). The number of insulin receptors (IR) in peripheral tissue is an important marker of insulin sensitivity. Diabetic rats showed a significantly reduced level of insulin receptors, as indicated by the results of western blot (Fig. S4.) and RT-PCR (Fig. S5). However, both mulberry leaf and glibenclamide treatments could significantly (P < 0.05) enhance insulin receptors expression.

Furthermore, NEFAs is antagonistic to adiponectin in obesity and type II diabetes^29,30^. Decreased adiponectin at transcription and post-translation levels was observed in diabetic rats, which was restored under mulberry leaf treatment (Fig. 4d and e). Adiponectin receptor 1 (ADPR1) expression of MTG was slightly elevated compared to the other groups, albeit it was not significant. Adiponectin receptor 2 (ADPR2) expression did not change between each group.

The results indicated that mulberry leaf reduced the NEFA signaling.

### Overall structural changes of intestinal microflora

**Figure 5:**
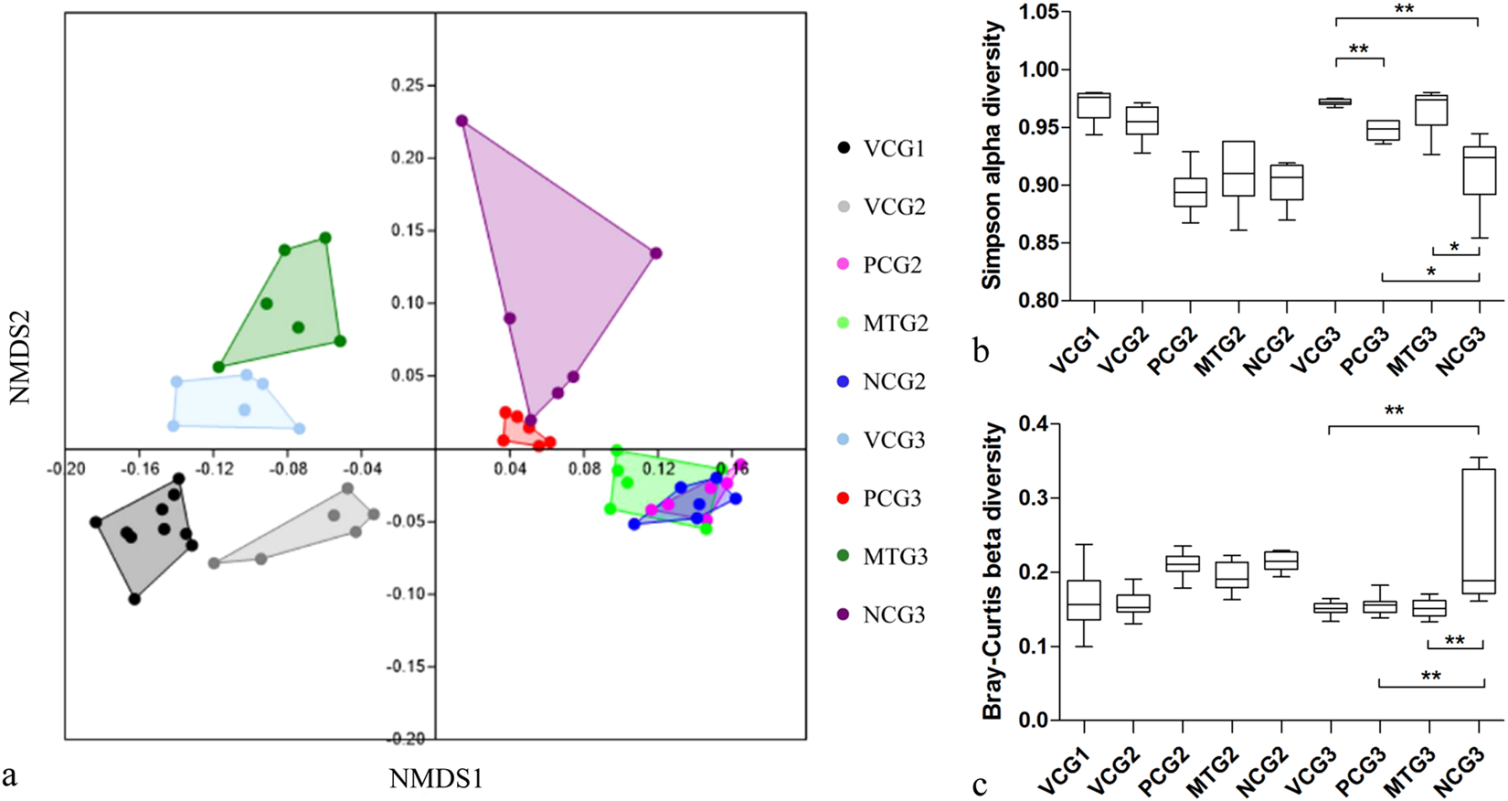
Effects of mulberry leaf on the community structure of intestinal microflora. (**a**) NMDS plot of the Bray–Curtis similarity coefficients calculated from the 16 S rRNA sequencing data of the gut microbiota; (**b**) Alpha diversity of each group revealed by the Simpson index. (**c**) Beta diversity of each group revealed by the Bray–Curtis index. *P < 0.05 between compared groups, **P < 0.01 between compared groups. Value = mean ± SD (N = 6).

An average of 22,825 clean reads were obtained per sample. After operational taxonomic unit (OTU)-based cluster and taxonomy annotation, these clean reads were classified into 139 taxa that were annotated at the genus or family level. The taxa annotated at the family level were labeled un-annotated genus (UAG) at the genus level (Supplementary Materials Table S15). In this annotated dataset, *Firmicutes* (accounting for 85.8%) and *Bacteroidetes* (accounting for 6.8%) were the most abundant phyla of the OTUs in all groups, in agreement with previous studies on the intestinal microbiota of rats^24^. Nonmetric multidimensional scaling (NMDS) plots of these data are shown in Fig. 5a. STZ treatment resulted in obvious divergences in the community structure of the intestinal microbiota between the VCG2 and diabetes mellitus groups (PCG2, MTG2 and NCG2). After 9 weeks of mulberry leaf treatment, MTG3 pose similar (ANOSIM, P = 0.099 > 0.05; α diversity, P = 0.471 > 0.05 and β diversity, P = 0.993 > 0.05) community structure of the intestinal microbiota with VCG3. However, there is a significant difference between VCG3 and NCG3 (ANOSIM, P = 0.004 < 0.05; α diversity, P = 0.0058 < 0.01 and β diversity, P = 0.0014 < 0.01). The Simpson and Bray–Curtis indices were used to indicate the α and β diversity (Fig. 5b and c), respectively. The calculation of the analysis of similarities (ANOSIM) confirmed the similarity and divergence. These results indicate that the mulberry leaf diet could improve the community structure of the intestinal microbiota of diabetes mellitus SD rats.

**Figure 6:**
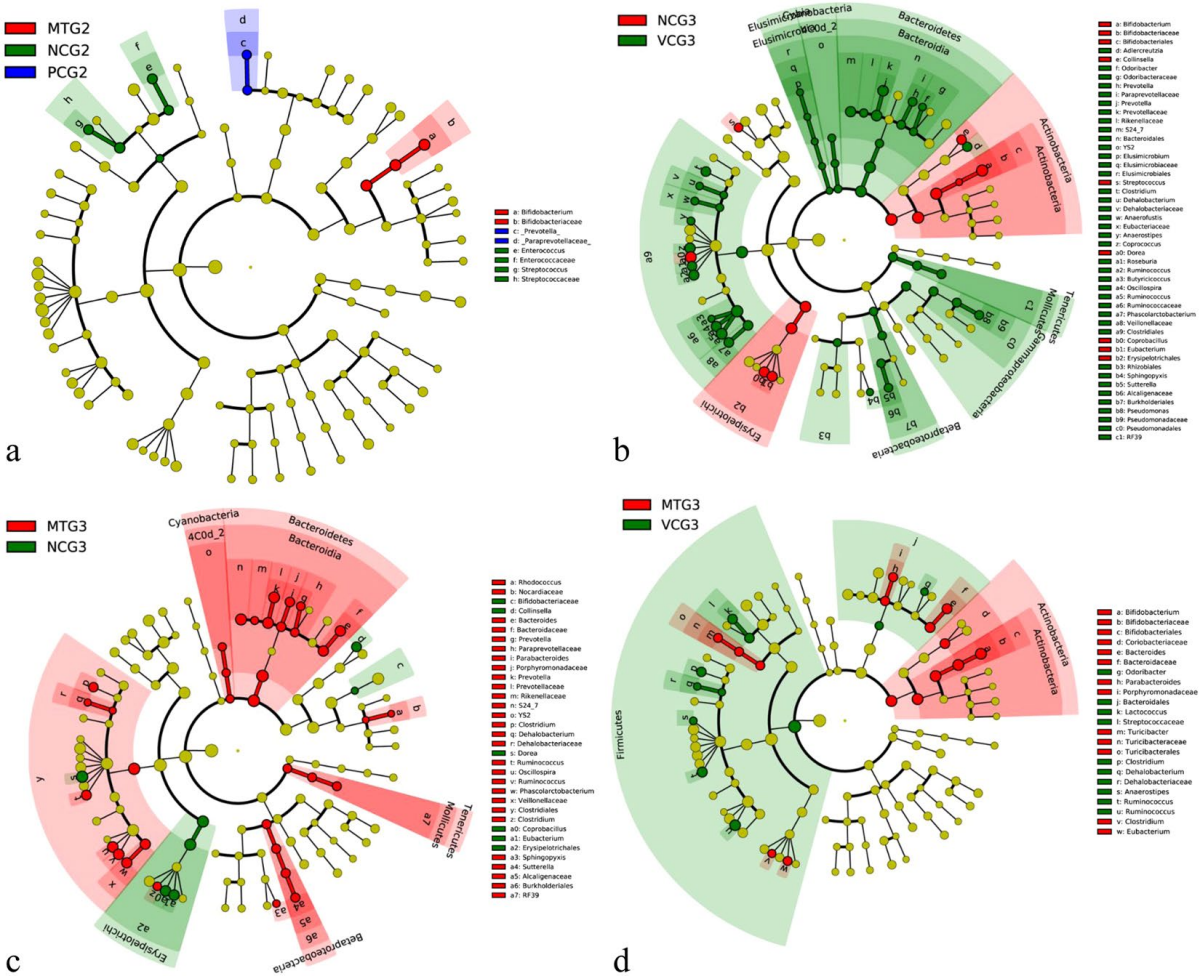
The structural changes of intestinal microflora affected by mulberry leaf according to the LEfSe analysis. (**a**) The similarity of intestinal microflora among STZ treatment groups (PCG2, MTG2, NCG2). (**b**) The changes of intestinal microflora between NCG3 and VCG3. (**c**) The changes of intestinal microflora between MTG3 and NCG3. (**d**) The changes of intestinal microflora between MTG3 and VCG3.

The changes in the specific taxa were then analyzed to determine the taxa that accounted for shifts in the community structure (Fig. 6) using the LEfSe^25^ online tool. As shown in Figs 5a and 6a, the STZ treatment groups (PCG2, MTG2, and NCG2) shared extremely similar (ANOSIM, P > 0.05) community structure. Compared with VCG3 at the phylum level, NCG3 increased the *Actinobacteria* and decreased the *Firmicutes*, *Bacteroidetes* and *Proteobacteria* levels. Diabetic rats posed remarkably elevated levels of *Bifidobacterium* (↑79-fold), *Collinsella* (↑5.7-fold), *Eubacterium* (↑19.9-fold), *Coprobacillus* (↑12.1-fold), *Dorea* (↑2.1-fold) and *Streptococcus* (↑4.9-fold) at the genus level compared with VCG3. However, mulberry leaf treatment could significantly reverse the influence of STZ treatment on intestinal microflora. At the genus level, *Bifidobacterium* (↓30.2%), *Collinsella* (↓85.7%), *Eubacterium* (↓81.5%), *Coprobacillus* (↓94.3%) and *Dorea* (↓67.4%) were significantly reduced in MTG compared with NCG3. At the family or genus levels, *Phascolarctobacterium* (↑6.2-fold), *Ruminococcus* (↑2.3-fold), *Oscillospira* (↑2.7-fold, *Ruminococcaceae* (↑1.5-fold), *Ruminococcus* (↑2.0-fold), *Clostridium* (↑3.2-fold) of *Firmicutes* and *S24-7* (↑4.6-fold), *Prevotella* (↑11.1-fold), *Parabacteroides* (↑2.7-fold), *Prevotella* (↑5.7-fold), *Bacteroides* (↑2.2-fold) of *Bacteroidetes* were significantly increased in MTG3 compared with NCG3. STZ-induced diabetic rats had significantly changed composition and function of the intestinal microbiota. Taken together, these data showed that mulberry leaf treatment reversed the majority of changed intestinal bacteria (Fig. 6c).

## Discussion

Hyperglycemia and insulin resistance in rats were successfully induced by single intravenous administration of STZ as a diabetogenic agent. Reed *et al*.^31^ fed SD rats a normal chow or high-fat diet (HFD) for 2 weeks and then injected them with STZ. Before STZ injection, FSI significantly increased in the HFD group (360 ± 36 pmol/L) compared with the normal chow group (178 ± 18 pmol/L). After STZ injection, FSI significantly decreased in the HFD group (186 ± 24 pmol/L), which was similar to the normal chow group. HFD feeding was likely to induce insulin resistance and increase the circulating insulin level. Although there was no variation in the FSI level between diabetes animals and vehicle control, decreased pancreas islet β-cell function and increased insulin resistance were observed in STZ-induced diabetes rats according to the HOMA. Glibenclamide is a traditional drug treating diabetes mainly by stimulating insulin release^32^, enhancing the insulin sensitivity of peripheral tissues^33^ and inhibiting liver glycogen decomposition and gluconeogenesis^34,35^. In the present study, glibenclamide significantly reduced FBG and HbA1c, and improved insulin sensitivity by increasing insulin receptors in peripheral tissue partially. Becknielsen *et al*.^33^ reported that treatment with glibenclamide could significantly increase insulin receptors of peripheral tissues in patients with type II diabetes. However, in general glibenclamide is not used to treat insulin resistance clinically. Mechanism of hypoglycemic effect of glibenclamide should be further studied. Clinically metformin is used to treat insulin resistance, primarily by suppressing hepatic gluconeogenesis^36^. In addition, metformin could increase insulin sensitivity, enhance peripheral glucose uptake^37^, decrease insulin-induced suppression of fatty acid oxidation^38^, and decrease absorption of glucose from the gastrointestinal tract^39^. STZ is always used in medical research to produce an animal model for hyperglycemia in a large single dose as well as type II diabetes or type I diabetes with multiple low doses. STZ-induced diabetes can be alleviated by promoting the secretion of insulin and/or improving insulin sensitivity. Therefore, both glibenclamide and metformin could be used as positive control treating STZ-induced diabetes. However, regardless of glibenclamide or metformin, their clinically therapeutic effect on diabetes are limited. Combined use of glibenclamide and metformin posed better therapeutic effect on diabetes clinically^40–42^, which is also suggested to be used as positive control in STZ-induced diabetes.

Mulberry leaf is reported to be anti-hyperglycemic, anti-hyperlipidemia, anti-obesity, antioxidant and anti-inflammatory^43–45^. The current study showed that mulberry leaf decreased the FBG and HbA1c, ameliorated the blood lipid levels and improved the insulin sensitivity in diabetic rats induced by STZ. The diabetic rats were characterized by weight loss, polydipsia, polyuria and polyphagia, which was consistent with a previous report^46^. Undoubtedly, mulberry leaf significantly improved the body weight, decreased the food and energy intake, and reduced the water intake and urination (clinical observation, data not shown) of diabetic rats. Mulberry leaf or its extract was rich in DNJ^47^, GABA, flavonoids, polyphenols^48^ and polysaccharide^49^. The isolated flavonol glycoside and polyphenol contents from mulberry leaves were shown to have antioxidant activity^43,44^ and be effective in attenuating body lipid accumulation and preventing obesity^45^. Moreover, long-term administration of ethanol extract (rich in flavonoids and polyphenols) from mulberry leaf had an anti-diabetic effect^50^ and restored arterial pressure^46^ in chronic diabetic rats. Mulberry DNJ, a potent glucosidase inhibitor, has been demonstrated as beneficial for suppressing abnormally high blood glucose levels, preventing diabetes^47^. Mulberry polysaccharides also played an important role in protecting against alloxan-induced pancreatic islet damage through their scavenging ability^49^. Mulberry leaf in this study was rich in polysaccharides, DNJ, flavonoid (rutin), polyphenols (chlorogenic acid and gallic acid) and GABA, which likely helped alleviate symptoms in STZ-induced diabetic rats.

In our study, we demonstrate that mulberry leaf alleviated diabetes partially by attenuating NEFA signaling. Insulin resistance is generally regarded as a pathological condition in which cells fail to respond to the normal actions of insulin, leading to high blood glucose. At the molecular level, cells sense insulin through insulin receptors, and the signal propagates through a cascade of molecules that are collectively known as the PI3K/Akt/mTOR signaling pathway^51^. The pathway’s sensitivity to insulin can be blunted by many factors, such as NEFAs, causing insulin resistance. Impaired β-cell function and insulin resistance could impair adipocyte metabolism, resulting in increased lipolysis and elevated NEFA levels. Elevations in both NEFAs and glucose can occur simultaneously; together, they are more deleterious to islet health and insulin action than either alone^16,52^. The present study showed that STZ-induced diabetic rats were strongly associated with insulin resistance and elevated circulating NEFAs levels. As an important factor in modulating insulin sensitivity, NEFAs can induce insulin resistance and impair β-cell function via PKC activation^53^.

Enhanced NEFA signaling has a negative effect on cellular energy homeostasis. NEFAs can be taken up from the blood by all cells that have mitochondria (mainly in liver and muscle). Increased circulating NEFAs reduce cellular glucose uptake and promote hepatic glucose output^13,27^. In addition, increased intracellular NEFAs result in competition with glucose for substrate oxidation, leading to a serial inhibition of pyruvate dehydrogenase, phosphofructokinase, hexokinase II and OXPHOS activity, ATP synthesis^28^. To further investigate the influence of mulberry leaf on NEFA signaling, key factors associated with energy metabolism were monitored. PGC-1α is a transcriptional coactivator that regulates the genes involved in mitochondrial fatty acid oxidation and ATP synthesis^54^. It is the major regulator of mitochondrial biogenesis and may also be involved in regulating cellular cholesterol homoeostasis and the development of obesity. Gene expression profiling studies have shown that decreased expression of PGC-1α and related gene products could affect mitochondrial function in people with insulin resistance and type II diabetes^55^. AK2, a phosphotransferase enzyme plays an important role in oxidative phosphorylation^56^, which is the most important metabolic pathway releasing energy that is used to reform ATP^57^. In addition, adiponectin is a protein hormone that modulates a number of metabolic processes^58^, including glucose regulation and fatty acid oxidation, and it plays a role in suppressing the metabolic derangements, which may result in obesity and type II diabetes^59^. In the present study, STZ-induced diabetic rats showed disordered energy homeostasis, which could be restored significantly under mulberry leaf treatment. These results indicated that mulberry leaf could reduce the NEFA signaling based on improving the cellular energy homeostasis.

Accumulating evidence has demonstrated that the changes in the composition of the intestinal microbiota may be specifically associated with hyperglycemia and diabetes^60–62^. In this study, the proportion of the phylum *Bacteroidetes* (↓64.9%) was significantly reduced, whereas the phyla *Firmicutes* (↓8.4%) and *Proteobacteria* (↓29.1%) were somewhat, but not significantly, reduced in the diabetic group. A reduction in the proportion of the phylum *Bacteroidetes* was always associated with obesity and hyperglycemia^61,62^. In an investigation of diabetic humans, the proportions of the phylum *Firmicutes* and class *Clostridia* are significantly reduced; the phyla *Bacteroidetes* and *Proteobacteria* were also reduced in the diabetic group and the proportion of *Betaproteobacteria* was positively correlated with the plasma glucose levels^63^. In a leptin-deficient ob/ob murine model, Ley *et al*.^61^ found a difference in the ratio of *Bacteroidetes* and *Firmicutes*, the two dominant intestinal bacterial phyla. Compared with their lean counterparts, obese mice showed a decrease in *Bacteroidetes* and corresponding increase in *Firmicutes*. When obese humans were placed on either a fat-restricted or carbohydrate-restricted low-calorie diet, an increase in the abundance of *Bacteroidetes* and a decrease in *Firmicutes* was reported^64^. In this study, mulberry leaf treatment significantly restored the phyla *Bacteroidetes* and *Proteobacteria* in STZ-induced diabetic rats. The proportion of *Firmicutes* was slightly, but not significantly, reduced; however, the class *Clostridia* was significantly increased after mulberry leaf treatment. Previous studies showed that the proportion of *Clostridia* in human adults with type II diabetes was significantly lower than in controls and showed a tendency to decrease with higher plasma glucose levels^63,65^. The restored phylum *Bacteroidetes* and *Proteobacteria* and class *Clostridia* might play important roles in ameliorating diabetes with mulberry leaf.

Most significantly, *Actinobacteria* was enriched by approximately 9.4-fold in diabetic rats compared with vehicle control, especially the genuses *Bifidobacterium* and *Collinsella*. Relative abundances of *Actinobacteria* were found in human adults with type II diabetes^63^. An increase in the gut *Actinobacteria* in obese people has also been observed in a core gut microbiome study of obese and lean twins^60^. However, several studies showed that the levels of *Bifidobacterim* and *Collinsella* were significantly and positively correlated with improved glucose tolerance and low-grade inflammation in prebiotic treated mice^66–68^. In this study, mulberry leaf treatment slightly decreased the level of *Bifidobacterim*, but it significantly reduced the proportion of *Collinsella*. The function of *Actinobacteria* in gut microbiota was not thoroughly understood. Therefore, further research is required to gain detailed information about gut *Actinobacteria* compositional changes and their associated impact with obesity and diabetes.

Hyperglycemia/diabetes has gained substantial attention worldwide as a serious public health issue. In the current study, mulberry leaf could reduce the FBG and HbA1c, ameliorate the blood lipid profile and improve the insulin sensitivity in diabetic rats induced by STZ. The abundant DNJ, flavonoids, polyphenols and polysaccharides in mulberry leaf may be responsible for the beneficial effect on diabetes. Mulberry leaf could alleviate diabetes through attenuating NEFA signaling based on improving the cellular energy homeostasis. In addition, mulberry leaf treatment restored the phyla *Bacteroidetes* and *Proteobacteria* and class *Clostridia*, which were associated with insulin resistance and diabetes. In summary, this report demonstrates for the first time that mulberry leaf could partially alleviate diabetes by attenuating NEFA signaling. In addition, the restored intestinal microflora communities contributed to the ameliorated diabetes. Mulberry leaf posed great potential in herbal remedy treating diabetes. However, the exact molecular mechanism underlying mulberry leaf-mediated diabetes alleviation remains to be further investigated.

## Methods

### Materials and component analysis

Mulberry leaves (*Morus alba var. multicaulis (Perrott.) Loud*.) were harvested from a farm in Danyang City, Jiangsu Province, China. The 4^th^ to 6^th^ leaves from the apex of healthy plants were plucked, thoroughly washed, dried at 45 °C for 36 h and ground to a fine powder in an electric grinder. The nutritional proximates (moisture, protein, fat, crude fiber, and ash), vitamins, minerals, fatty acids and polysaccharide of the powder were measured using validated standard methods (Supplementary Materials Table S1).

Functional components, including DNJ, rutin, chlorogenic acid, gallic acid, β-sitosterol, γ-aminobutyric acid (GABA) and carotene, of the mulberry leaf powder were detected by high-performance liquid chromatography (HPLC) with an Agilent 1100 HPLC system (Agilent Technologies Inc., California, USA) together with a diode array detector. All standard substances (purity > 98%) were purchased from Beijing Solarbio Science & Technology Co., Ltd. (Beijing, China).

The rodent diets included a high-fat diet (45% energy provided by fat) (HFD), HFD normal chow (80% w/w) with mulberry leaf powder (20% w/w) diet (HFMD) and AIN93G diet^50^ as a vehicle control, which were prepared by Beijing HFK Bioscience Co. Ltd. (Beijing, China). All diets were vacuum packed and sterilized.

### Animal experimental design

All experimental procedures and use of animals were conducted according to the Guide for the Care and Use of Laboratory Animals published by the US National Institutes of Health and approved by Animal Ethics Committee of China Agricultural University, Beijing (the approval ID of this study is KY150009). Five-week-old Sprague-Dawley male rats free from specific pathogens were obtained from Vital River Laboratories Inc. (Beijing, China). After acclimatization for one week, healthy rats were fed a HFD or AIN93G diet. After 4 weeks, the rats fed a HFD were rendered diabetic by a single i.p. administration of streptozotocin (STZ) (sigma, USA) (35 mg/kg B.W.) via tail intravenous injection. After 72 h of injection, the fasting blood-glucose levels (16 h) were detected with a glucometer (ACCU-CHEK per forma, Roche, USA). The animals with blood glucose levels over 7.8 mmol/L were considered diabetes rats. The diabetic rats were randomly distributed into 3 experimental groups (6 rats/group) considering the body weights and blood glucose levels with a stratified randomization procedure. The negative control group (NCG) was fed a HFD, positive control group (PCG) was fed a HFD and glibenclamide (2.5 mg/kg B.W. every day), mulberry leaf treatment group (MTG) was fed a HFMD, and vehicle control group (VCG) was fed a AIN93G diet^69^.

The animals were housed (3 rats/cage) in a specific pathogen-free (SPF) animal laboratory of the Supervision & Testing Center for GMOs Food Safety, Ministry of Agriculture (Beijing, China) with the license number SYXK (Beijing) 2010–0036 under standard laboratory conditions (adequate fresh air exchange, temperature 20–24 °C and relative humidity 40–70%). A 12-h light/dark automatic cycle of artificial illumination was used. All animals were provided sterile drinking water.

### Biochemical and clinical assays

Daily food consumption was determined by subtracting the remaining feed from the feed provided to the rats. The body weights were recorded once a week and clinical behavior observation was conducted daily during the entire experiment. The blood glucose concentration was determined after overnight fasting (16 h). The glucose tolerance test (GTT) was performed on the 12^th^ week before the rat was killed. Briefly, the rats were intraperitoneally injected with glucose (2.0 g/kg BW, sigma) and then examined using a blood glucose meter (Accu-Chek® Performa, Roche) at 0, 15, 30, 60, 90, and 120 min post injection. All rats were sacrificed by decollation after anesthesia utilizing chloral hydrate, and their blood was collected from the inner canthus. Gross necropsy of the major organs was conducted by visual inspection. Pancreas islet tissues were fixed in 10% formalin solution and the pathological sections were H&E stained and observed by a microscope (Germany, Leica DM2500).

Glycosylated hemoglobin (HbA1c) was measured utilizing a VARIANT^TM^ II Hemoglobin test system (Bio-Rad, USA). Hematochemistry analyses for alanine aminotransferase (ALT), aspartate aminotransferase (AST), alkaline phosphatase (ALP), triglyceride (TG), total cholesterol (CHO), high density lipoprotein (HDL), low density lipoprotein (LDL), and NEFAs were performed using an RA-1000 autoanalyzer (Technicon, Tarrytown, NY, USA). The serum insulin level was measured with an Insulin (rat) EIA Kit (Cayman, USA) according to the instructions.

### RT-PCR analysis

Total RNA was isolated from liver or epididymis white fat tissue grinding fluid using Trizol reagent (TIANGEN, China). Complementary DNA was synthesized using a Quantscript RT Kit (TIANGEN, China) and the products were used as template for RT-PCR. The primers used are shown in the Supplementary Materials Table S2. Real-time PCR was performed on an iCycler Thermal Cycler PCR System (Bio-Rad Laboratories, Hercules, USA). The following general RT-PCR protocol was used: denaturation program (95 °C for 5 min); a three-segment amplification program repeated 40 times (95 °C for 30 s, annealing temperature for 45 s and 72 °C for 45 s); and extension (72 °C for 10 min). All samples were conducted in triplicate. Data acquisition and subsequent data analyses were performed using Quantity One 1-D Analysis Software (Bio-Rad).

### Western blot analysis

The liver and subcutaneous fat were used for protein isolation and quantification; tissues were solubilized in RIPA Lysis Buffer (50 mM Tris (pH 7.4), 0.15 M NaCl, 1% Triton X-100, 1% sodium deoxycholate, 0.1% SDS, 0.1 M EDTA, 2 mg/L leupeptin, and 100 mg/L sodium fluoride). Protein was isolated by centrifugation (4 °C, 12000 g, 15 min), and the content was determined by the Bio-Rad dye-binding assay; 50 μg of protein was then separated by 12% SDS-PAGE gel. The proteins were subsequently transferred to PVDF using the Mini-PROTEAN^®^ Tetra Vertical Electrophoresis Cell (Bio-Rad Laboratories) (80 V, 120 min), and the filters were incubated for 1.5 h in Blotto solution (5% milk powder (w/v) in TBST and 3.2 mM MgCl_2_; pH 7.4). The unwashed filters were then incubated with primary antibodies (rabbit anti-rat PKC α + β + γ antibody, mouse anti-rat adiponectin antibody, total OXPHOS rodent WB antibody and anti-PGC1 alpha antibody) (Abcam, Germany) (1:1000 dilution) in Blotto solution at 4 °C for 16 h. The filters were then washed 3 times in TBST before incubation with secondary anti-rabbit or anti-mouse antibody (1:2000 dilution) tagged with horseradish peroxidase (HRP). The filters were washed again. Western blot detection was performed using chemiluminescent HPR substrate (Millipore Corporation, Billerica, USA) and subsequent autoradiography was performed with a MiniChemi^TM^ 610 Mini Chemiluminescent Imaging and Analysis System (Beijing Sage Creation Science Co., Ltd. China). The products were quantified by densitometry using the ImageJ software. Each blot was performed in triplicate.

### Intestinal microflora analysis

Feces from rats were collected at three time points (after environmental adaptation, after STZ administration, and 60 days after mulberry leaf treatment) and were stored at −80 °C until use. Six samples from each group were used for the intestinal microbiota analysis. Microbial genomic DNA was extracted from each fecal sample (0.1 g) using a previously described method^70^. The V3 + V4 region of the 16 S rRNA was amplified by PCR and sequenced using a HiSeq platform (Illumina, San Diego, CA, USA) at Novogene Bioinformatics Institute (Beijing, China). Sequences analysis was performed using Uparse software (Uparse v7.0.1001)^71^. Sequences with ≥97% similarity were assigned to the same operational taxonomic unit (OUT). Taxonomic annotation was conducted using a RDP classifier (Version 2.2)^72^. Alpha diversity is applied in analyzing the complexity of species diversity for each group with the diversity/diversity-indices of the PAST version 2.17 software program. Beta diversity analysis was used to evaluate differences in the species complexity in samples. Cluster analysis was preceded by principal component analysis. Community structure variance analysis was conducted with LEfSe software using the default parameters (http://huttenhower.sph.harvard.edu/galaxy/)^73^.

### Statistical analysis

The tabulated data are presented in the form of the mean value and standard deviation (SD) calculated with IBM SPSS Statistics 20.0 software (IBM SPSS Inc., USA). A one-way analysis of variance (ANOVA) was used to evaluate the homogeneity variance for each group. SPSS was used to perform the multiple comparisons between the tested groups (Dunnett’s test). Statistically significant differences were defined as p < 0.05.

## Electronic supplementary material

Supplementary materials

## References

[1] IDF. International Diabetes Federation Diabetes Atlas Seventh Edition 2015. *International Diabetes Federation*. Seventh Edition, 9–12 (2016).

[2] Mckimmie RL (2008). Hepatic steatosis and subclinical cardiovascular disease in a cohort enriched for type 2 diabetes: the Diabetes Heart Study. Am J Gastroenterol..

[3] Kitabchi AE, Umpierrez GE, Miles JM, Fisher JN (2009). Hyperglycemic crises in adult patients with diabetes. Diabetes care..

[4] Wang C (2016). Increased serum microRNAs are closely associated with the presence of microvascular complications in type 2 diabetes mellitus. Sci Rep..

[5] Carlson RW (2001). Manual of Intensive Care Medicine. Jama J Am Med Assoc..

[6] De Smet PAGM (2002). Drug therapy: Herbal remedies. New Engl J Med.

[7] Jouad H, Haloui M, Rhiouani H, Hilaly JE, Eddouks M (2001). Ethnobotanical survey of medicinal plants used for the treatment of diabetes, cardiac and renal diseases in the North centre region of Morocco (Fez–Boulemane). J Ethnopharmacol..

[8] Asano N (2001). Polyhydroxylated alkaloids isolated from mulberry trees (Morus alba L.) and silkworms (Bombyx mori L.). J Agr Food Chem..

[9] Venkatesh Kumar R, Chauhan S (2008). Mulberry: life enhancer. J Med Plants Res..

[10] Kimura T (2004). Determination of 1-deoxynojirimycin in mulberry leaves using hydrophilic interaction chromatography with evaporative light scattering detection. J Agr Food Chem..

[11] Reaven GM, Hollenbeck C, Jeng C-Y, Wu MS, Chen Y-DI (1988). Measurement of plasma glucose, free fatty acid, lactate, and insulin for 24 h in patients with NIDDM. Diabetes..

[12] Boden G (1997). Role of fatty acids in the pathogenesis of insulin resistance and NIDDM. Diabetes..

[13] Roden M (1996). Mechanism of free fatty acid-induced insulin resistance in humans. J Clin Invest..

[14] Santomauro A (1999). Overnight lowering of free fatty acids with Acipimox improves insulin resistance and glucose tolerance in obese diabetic and nondiabetic subjects. Diabetes..

[15] Dobbins RL (1998). A fatty acid-dependent step is critically important for both glucose-and non-glucose-stimulated insulin secretion. J Clin Invest..

[16] Prentki M, Joly E, El-Assaad W, Roduit R (2002). Malonyl-CoA Signaling, Lipid Partitioning, and Glucolipotoxicity Role in β-Cell Adaptation and Failure in the Etiology of Diabetes. Diabetes..

[17] Guo Z (2016). Intestinal Microbiota Distinguish Gout Patients from Healthy Humans. Sci Rep..

[18] Matsumoto M (2011). Impact of Intestinal Microbiota on Intestinal Luminal Metabolome. Sci Rep..

[19] Karlsson FH (2013). Gut metagenome in European women with normal, impaired and diabetic glucose control. Nature..

[20] Sepp E, Kolk H, Lõivukene K, Mikelsaar M (2014). Higher blood glucose level associated with body mass index and gut microbiota in elderly people. Microb Ecol Health Dis..

[21] Arimatsu K (2013). Oral pathobiont induces systemic inflammation and metabolic changes associated with alteration of gut microbiota. Sci Rep..

[22] Poroyko VA (2016). Chronic Sleep Disruption Alters Gut Microbiota, Induces Systemic and Adipose Tissue Inflammation and Insulin Resistance in Mice. Sci Rep..

[23] Khosravi Y (2015). Helicobacter pylori infection can affect energy modulating hormones and body weight in germ free mice. Sci Rep..

[24] Zhang X (2012). Structural changes of gut microbiota during berberine-mediated prevention of obesity and insulin resistance in high-fat diet-fed rats. PloS one..

[25] Paulson JN, Stine OC, Bravo HC, Pop M (2013). Differential abundance analysis for microbial marker-gene surveys. Nat Methods..

[26] Rosen ED, Spiegelman BM (2006). Adipocytes as regulators of energy balance and glucose homeostasis. Nature..

[27] Roden M (2000). Effects of free fatty acid elevation on postabsorptive endogenous glucose production and gluconeogenesis in humans. Diabetes.

[28] Simha V, Garg A (2006). Lipodystrophy: lessons in lipid and energy metabolism. Curr Opin Lipidol..

[29] Gil-Campos M, Ramírez Tortosa MC, Aguilera CM, Cañete R, Gil A (2011). Fasting and postprandial adiponectin alterations anticipate NEFA and TNF-α changes in prepubertal obese children. Nutr Metab Cardiovasc Dis..

[30] Reddy NL (2014). Enhanced thermic effect of food, postprandial NEFA suppression and raised adiponectin in obese women who eat slowly. Clin Endocrinol..

[31] Reed MJ (2000). A new rat model of type 2 diabetes: the fat-fed, streptozotocin-treated rat. Metab Clin Exp..

[32] Serrano-Martín X, Payares G, Mendoza-León A (2006). Glibenclamide, a blocker of K + (ATP) channels, shows antileishmanial activity in experimental murine cutaneous leishmaniasis. Antimicrob Agents Ch..

[33] Beck-Nielsen H, Pedersen O, Lindskov HO (1979). Increased insulin sensitivity and cellular insulin binding in obese diabetics following treatment with glibenclamide. Acta Endocrinol..

[34] Carvalho-Martini M, de Oliveira DS, Suzuki-Kemmelmeier F, Bracht A (2006). The action of glibenclamide on glycogen catabolism and related parameters in the isolated perfused rat liver. Res Commun Mol Path..

[35] Okamoto RM (2007). Comparison with Glimepiride and Glibenclamide on Gycogen Accumulation in Liver. Diabetes..

[36] Kirpichnikov D, McFarlane SI, Sowers JR (2002). Metformin: an update. Ann Intern Med..

[37] Zhou G (2001). Role of AMP-activated protein kinase in mechanism of metformin action. J Clin Invest..

[38] Collier CA, Bruce CR, Smith AC, Lopaschuk G, Dyck DJ (2006). Metformin counters the insulin-induced suppression of fatty acid oxidation and stimulation of triacylglycerol storage in rodent skeletal muscle. Am J Physiol-Endoc M..

[39] Rena G, Pearson ER, Sakamoto K (2013). Molecular mechanism of action of metformin: old or new insights?. Diabetologia..

[40] González-Ortiz M (2009). Efficacy of glimepiride/metformin combination versus glibenclamide/metformin in patients with uncontrolled type 2 diabetes mellitus. J Diabetes Complica..

[41] Tosi F (2003). Combination treatment with metformin and glibenclamide versus single-drug therapies in type 2 diabetes mellitus: a randomized, double-blind, comparative study. Metabolism.

[42] Barelli, G. & De Regis, M. Glibenclamide-metformin combination for the treatment of diabetes mellitus of type II. *U.S. Patent* No. 5,922,769. (1999).

[43] Katsube T (2006). Antioxidant flavonol glycosides in mulberry (Morus alba L.) leaves isolated based on LDL antioxidant activity. Food Chem..

[44] Katsube T, Tsurunaga Y, Sugiyama M, Furuno T, Yamasaki Y (2009). Effect of air-drying temperature on antioxidant capacity and stability of polyphenolic compounds in mulberry (Morus alba L.) leaves. Food Chem..

[45] Wu C-H (2010). Improvement in high-fat diet-induced obesity and body fat accumulation by a Nelumbo nucifera leaf flavonoid-rich extract in mice. J Agr Food Chem..

[46] Naowaboot J (2009). Mulberry leaf extract restores arterial pressure in streptozotocin-induced chronic diabetic rats. Nutr Res..

[47] Kimura T (2007). Food-grade mulberry powder enriched with 1-deoxynojirimycin suppresses the elevation of postprandial blood glucose in humans. J Agr Food Chem..

[48] Dugo P (2009). Characterization of the polyphenolic fraction of Morus alba leaves extracts by HPLC coupled to a hybrid IT‐TOF MS system. J Sep Sci..

[49] Li Y-G (2011). Hybrid of 1-deoxynojirimycin and polysaccharide from mulberry leaves treat diabetes mellitus by activating PDX-1/insulin-1 signaling pathway and regulating the expression of glucokinase, phosphoenolpyruvate carboxykinase and glucose-6-phosphatase in alloxan-induced diabetic mice. J Ethnopharmacol..

[50] Naowaboot J, Pannangpetch P, Kukongviriyapan V, Kongyingyoes B (2009). Antihyperglycemic, antioxidant and antiglycation activities of mulberry leaf extract in streptozotocin-induced chronic diabetic rats. Plant Food Hum Nutr..

[51] Wang G (2010). Singularity analysis of the AKT signaling pathway reveals connections between cancer and metabolic diseases. Phys Biol..

[52] Leung N (2004). Prolonged increase of plasma non-esterified fatty acids fully abolishes the stimulatory effect of 24 hours of moderate hyperglycaemia on insulin sensitivity and pancreatic beta-cell function in obese men. Diabetologia..

[53] Shulman GI (2000). Cellular mechanisms of insulin resistance. J Clin Invest..

[54] Shi Y (2016). Resveratrol enhances HBV replication through activating Sirt1-PGC-1α-PPARα pathway. Sci Rep..

[55] Patti ME (2003). Coordinated reduction of genes of oxidative metabolism in humans with insulin resistance and diabetes: Potential role of PGC1 and NRF1. PNAS..

[56] Köhler C (1999). Release of adenylate kinase 2 from the mitochondrial intermembrane space during apoptosis. Febs Lett..

[57] Marini C (2016). Divergent targets of glycolysis and oxidative phosphorylation result in additive effects of metformin and starvation in colon and breast cancer. Sci Rep..

[58] Mori T (2014). Ultrastructural Localization of Adiponectin protein in Vasculature of Normal and Atherosclerotic mice. Sci Rep..

[59] Ukkola O, Santaniemi M (2002). Adiponectin: a link between excess adiposity and associated comorbidities?. J Mol Med..

[60] Turnbaugh PJ (2009). A core gut microbiome in obese and lean twins. Nature..

[61] Ley RE (2005). Obesity alters gut microbial ecology. PNAS..

[62] Turnbaugh PJ (2006). An obesity-associated gut microbiome with increased capacity for energy harvest. Nature..

[63] Larsen N (2010). Gut microbiota in human adults with type 2 diabetes differs from non-diabetic adults. PloS one..

[64] Ley RE, Turnbaugh PJ, Klein S, Gordon JI (2006). Microbial ecology: human gut microbes associated with obesity. Nature..

[65] Wu X (2010). Molecular characterisation of the faecal microbiota in patients with type II diabetes. Curr Microbiol..

[66] Cani PD (2009). Changes in gut microbiota control inflammation in obese mice through a mechanism involving GLP-2-driven improvement of gut permeability. Gut..

[67] Cani PD (2008). Changes in gut microbiota control intestinal permeability-induced inflammation in obese and diabetic mice through unexpected dependent mechanisms. Diabetologia..

[68] Greetham HL (2004). Allobaculum stercoricanis gen. nov., sp. nov., isolated from canine feces. Anaerobe..

[69] Reeves PG, Nielsen FH, Fahey Jr GC (1993). AIN-93 purified diets for laboratory rodents: final report of the American Institute of Nutrition ad hoc writing committee on the reformulation of the AIN-76A rodent diet. J nutr..

[70] Guo M (2014). Combination of metagenomics and culture-based methods to study the interaction between ochratoxin a and gut microbiota. Toxicol Sci..

[71] Edgar RC (2013). UPARSE: highly accurate OTU sequences from microbial amplicon reads. Nat Methods..

[72] Wang Q, Garrity GM, Tiedje JM, Cole JR (2007). Naive Bayesian classifier for rapid assignment of rRNA sequences into the new bacterial taxonomy. Appl Environ Microb..

[73] Segata N (2011). Metagenomic biomarker discovery and explanation. Genome Biol..

