# Supplementary material for: Mulberry leaf alleviates streptozotocin-induced diabetic rats by attenuating NEFA signaling and modulating intestinal microflora
Source: Sci Rep. 2017 Sep 21;7:12041. doi: 10.1038/s41598-017-12245-2 (PMC5608946; doi:10.1038/s41598-017-12245-2)
Supplement: Supplementary file 1 — Supplementary materials [file 41598_2017_12245_MOESM1_ESM.pdf]

Mulberry leaf alleviates streptozotocin-induced diabetic rats by attenuating NEFA signaling and modulating intestinal microflora

Yao Sheng[a], Shujuan Zheng[a], Tianshi Ma[a], Chuanhai Zhang[a], Xiaoqun Ou[a], Xiaoyun He[a,b], Wentao Xu[a,b], Kunlun Huang[a,b*]

[a] Beijing Advanced Innovation Center for Food Nutrition and Human Health, College of Food Science and Nutritional Engineering, China Agricultural University, Beijing, China, 100083.

[b] Key Laboratory of Safety Assessment of Genetically Modified Organism (Food Safety), Ministry of Agriculture, Beijing, China, 100083.

Running Title: Mulberry leaf alleviates diabetes by reducing NEFAs and restoring intestinal microbiota communities

[*]Corresponding author:

Dr. Kunlun Huang

College of Food Science and Nutritional Engineering, China Agricultural University, No. 17 Tsinghua East Road Beijing, China, 100083.

Fax/Phone: (8610)62738552; E-mail: foodsafety66@cau.edu.cn

Table S1. Methods for Composition Analysis

Composition parameters	**Method**
Moisture	Chinese standard GB 5009.3-2010. 2010. National food safety standard: Determination of moisture in foods. Standards Press of China, Beijing, China.
Ash	Chinese standard GB 5009.4-2010. 2010. National food safety standard: Determination of ash in foods. Standards Press of China, Beijing, China.
Protein	Chinese standard GB 5009.5-2010. 2010. National food safety standard: Determination of protein in foods. Standards Press of China, Beijing, China.
Crude Fiber	Chinese standard GB/T 5009.10-2003. 2003. National food safety standard: Determination of crude fiber in vegetable foods. Standards Press of China, Beijing, China.
Fat	Chinese standard GB/T 5009.6-2003. 2003. National food safety standard: Determination of fat in foods. Standards Press of China, Beijing, China.
Calcium	Chinese standard GB/T 5009.92-2003. 2003. National food safety standard: Determination of calcium in foods. Standards Press of China, Beijing, China.
Zinc	Chinese standard GB/T 5009.91-2003. 2003. National food safety standard: Determination of zinc in foods. Standards Press of China, Beijing, China.
Copper	Chinese standard GB/T 5009.13-2003. 2003. National food safety standard: Determination of copper in foods. Standards Press of China, Beijing, China.
Manganese	Chinese standard GB/T 5009.90-2003. 2003. National food safety standard: Determination of iron,magnesium and manganese in foods. Standards Press of China, Beijing, China.
Potassium	Chinese standard GB/T 5009.91-2003. 2003. National food safety standard: Determination of potassium and sodium in foods. Standards Press of China, Beijing, China.
Phosphorus	Chinese standard GB/T 5009.87-2003. 2003. National food safety standard: Determination of phosphorus in foods. Standards Press of China, Beijing, China.
Vitamin E	Chinese standard GB/T 5009.82-2003. 2003. National food safety standard: Determination of retinol and tocopherol in foods. Standards Press of China, Beijing, China.
Vitamin B1	Chinese standard GB 5413.11-2010. 2010. National food safety standard: Determination of vitamin B1 in foods for infants and young children,milk and milk products. Standards Press of China, Beijing, China.
Vitamin B2	Chinese standard GB 5413.12-2010. 2010. National food safety standard: Determination of vitamin B2 in foods for infants and young children,milk and milk products. Standards Press of China, Beijing, China.
Fatty acids	Chinese standard GB/T 21514-2008. 2008. Determination of fatty acids in feeds. Standards Press of China, Beijing, China.
Polysaccharides	Chinese standard NY/T 1676-2008. 2008.National food safety standard:

	Determination of crude mushroom polysaccharide s. Standards Press of China, Beijing, China.

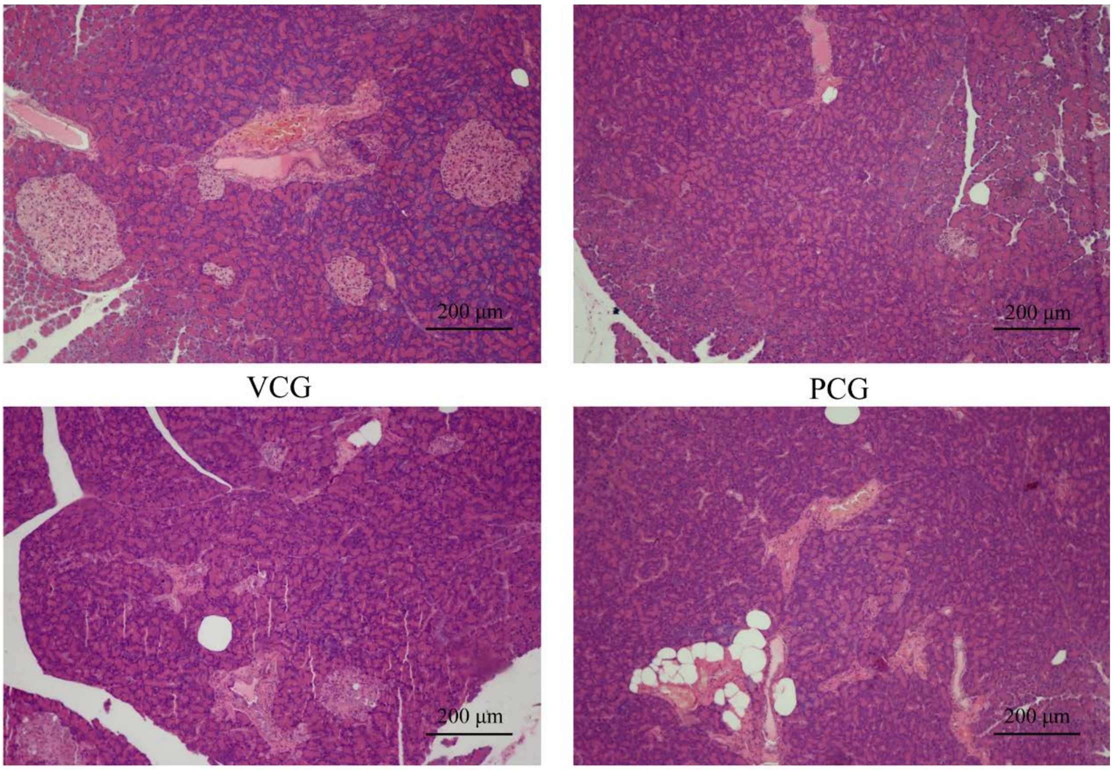

VCG PCG

MTG NGG

Figure S1. Histological data on pancreas (H & E stain). Diabetic rat induced by STZ posed significantly damaged pancreas islet as showed in figure S1 (NCG, PCG and MTG). The number and size of pancreas islet were decreased; mass of necrocytosis were observed in pancreas islet β-cell; in addition, vacuolization was found in pancreas in NCG. However, mulberry leaf treatment could inhibit necrocytosis and vacuolization indicating beneficial effect in protecting pancreas and pancreas islet damaged by STZ.

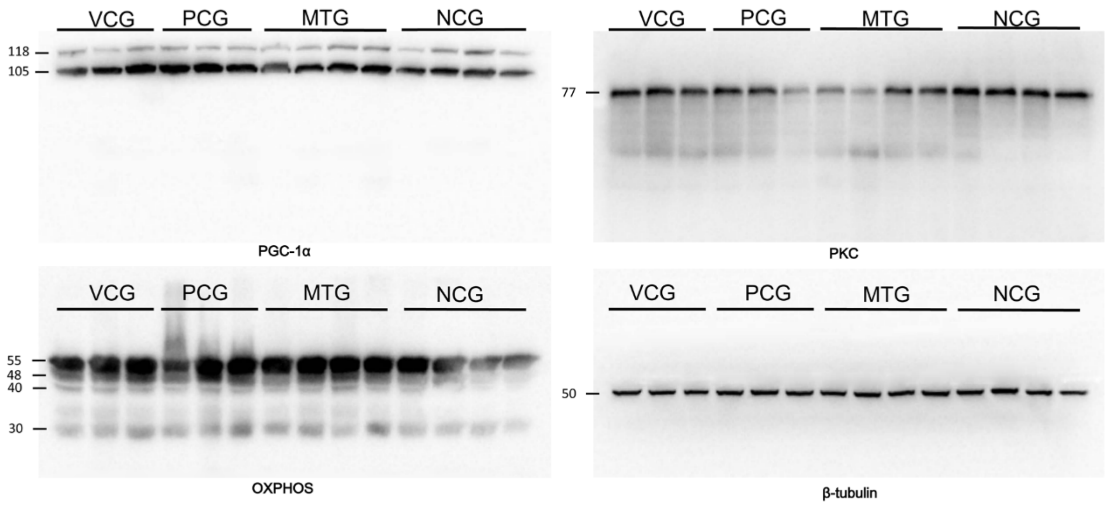

Figure S2. Western blot for protein in liver tissue, mulberry leaf decreased PKC and increased OXPHOS expression. β-tubulin serves as a loading control. For presentation purposes additional lanes were excised. PKC, PGC-1α, OXPHOS and β-tubulin were cropped from different parts of the same gel. These proteins were stained one by one after using Restore™ Western Blot Stripping Buffer (Thermo Scientific, USA).

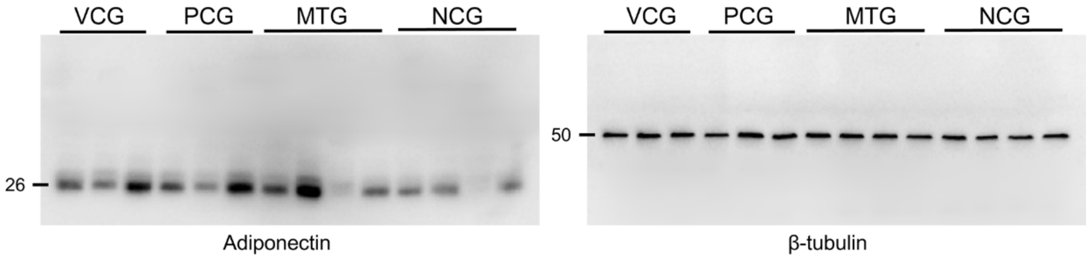

Figure S3. Western blot for protein in subcutaneous fat, mulberry leaf increased adiponectin expression in subcutaneous fat. β-tubulin serves as a loading control. For presentation purposes additional lanes were excised. Adiponectin and β-tubulin were cropped from different parts of the same gel. Both proteins were stained one by one after using Restore™ Western Blot Stripping Buffer (Thermo Scientific, USA).

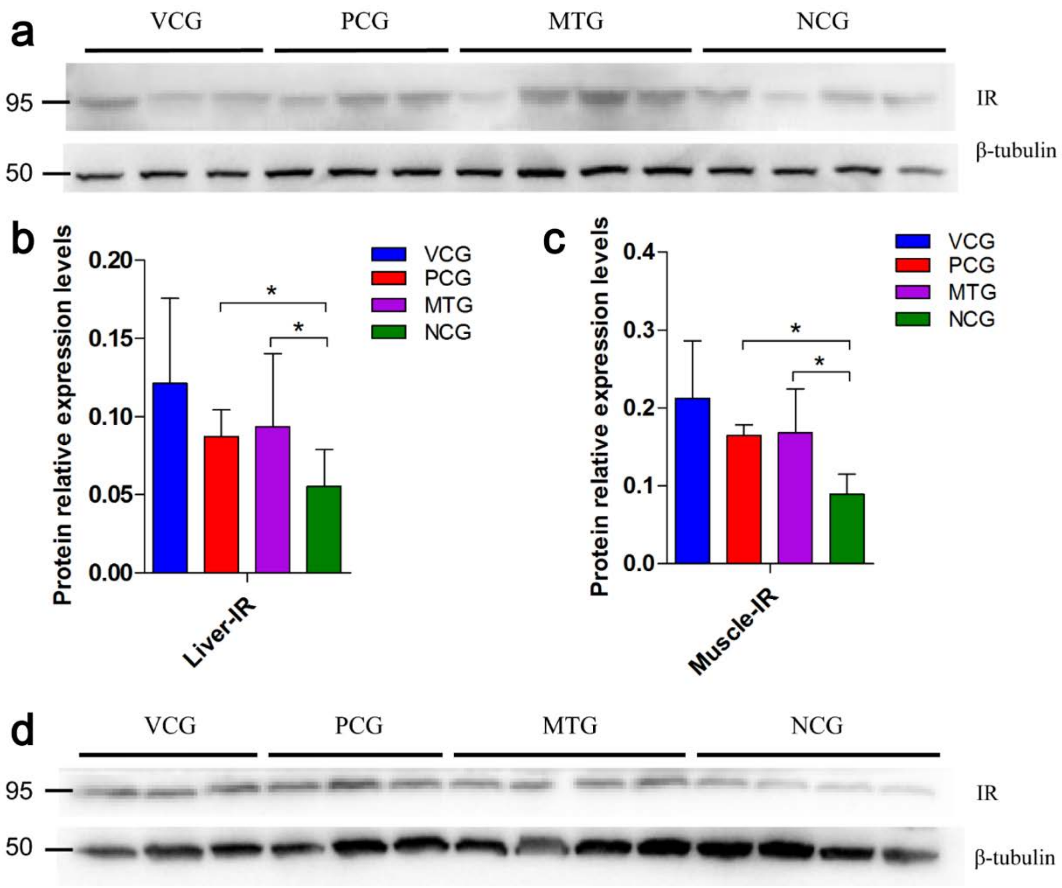

Figure S4. Insulin receptors (IR) expression in peripheral tissue. (**a, b**) Western blot for insulin receptor (IR) in liver tissue. (**c, d**) Western blot for insulin receptor (IR) in liver tissue. IR was significantly ($P < 0.05$) up-regulated in peripheral tissue (liver and muscle) of both mulberry leaf and glibenclamide treatments groups at post-translation level. β-tubulin serves as a loading control. For presentation purposes additional lanes were excised. IR and β-tubulin were cropped from different parts of the same gel. Both proteins were stained one by one after using Restore™ Western Blot Stripping Buffer (Thermo Scientific, USA). * $P < 0.05$ compared with NCG. Value=mean ± SD (N=3-4).

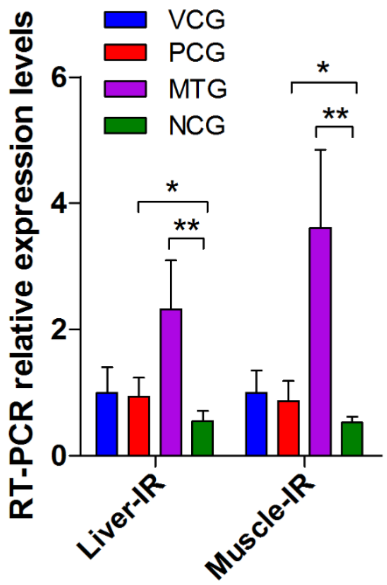

Figure S5, IR expression on RT-PCR, IR was significantly up-regulated in peripheral tissue (liver and muscle) of both mulberry leaf ($P < 0.01$) and glibenclamide ($P < 0.05$) treatments groups at transcription level. * $P < 0.05$ compared with NCG, ** $P < 0.01$ compared with NCG. Value=mean ± SD (N=6).

Table S2. The primers used in reverse transcription-PCR analysis

Gene	Primer sequences (forward)	Primer sequences (Reverse)	Amplicon length (bp)	TM (°C)
Adiponectin	CTACTGTTGCAAGCTCTCC	CTTCACATCTTTCATGTACACC	513	55
ADPR1	CTTCTACTGCTCCCCACAGC	TCCCAGGAACACTCCTGCTC	139	60
ADPR2	TGTAAGGTGTGGGAAGGTCG	GGAAAGAAGGCATAGGAGGC	112	58
PGC-1α	AGTTTTTGGTGAAATTGAGGAAT	TCATACTTGCTCTTGGTGGAAGC	249	58
AK2	GTGACCTTGCTGTCTGAGAA	TCCGCTTGTGGAATACTACA	240	57
PKC1	CAACCAAGAGGAGGGTGAAT	CACTGTCTGGAAGCAGGAGT	397	58
IR	GGCGAGAAGACCATTGATT	AAGTGACACCAGAGCATAGGA	189	58
β-actin	GTCAGGTCATCACTATCGGCAAT	AGAGGTCTTTACGGATGTCAACGT	147	60

Table S3. Nutritional proximates, vitamins, minerals of mulberry leaf powder. Value=mean ± SD (N=3)

Substance	Concentration
Protein (%)	19.6±1.6
Crude fibre (%)	11.200±0.889
Lipid (%)	1.780±0.153
Vitamin b1 (mg/100g)	0.002±0
Vitamin b2 (mg/100g)	0.738±0.082
Moisture (%)	9.460±0.681
Ash (%)	9.480±0.549
Kalium (mg/kg)	12060±1086
Calcium (mg/kg)	16800±8075
Sodium (mg/kg)	133.8±9.81
Magnesium (mg/kg)	5201±386
Ferrum (mg/kg)	316.7±27.5
Manganese (mg/kg)	44.5±2.86
Zinc (mg/kg)	30.6±1.46
Cuprum (mg/kg)	9.6±0.56
Phosphorus (mg/kg)	4138±126

Table S4. Fatty acids of mulberry leaf powder. Value=mean ± SD (N=3)

Fatty acids	Percentage (%)
C14:0	0.46±0.021
C16:0	18.12±1.26
C16:1	0.12±0.007
C17:0	0.28±0.011
C18:0	3.69±0.165
C18:1	3.00±0.196
C18:2	23.00±1.83
C18:3	39.31±2.06
C20:0	1.54±0.061
C22:0	1.16±0.087
Others	9.32±0.646

Table S5. Body weight (g) changes of all animals. Value=mean ± SD (N=6)

weeks	0	1	2	3	4	5	6	7	8	9	10	11	12	13
VCG	155±12	219±15	285±22	340±26	364±26	375±28	398±31[a]	419±33[a]	440±41[a]	463±44[a]	483±46[a]	498±47[a]	512±45[a]	523±51[a]
PCG	155±9	197±10	256±18	319±22	359±25	338±34	342±58[ab]	360±48[ab]	377±52[ab]	383±55[ab]	387±64[b]	395±68[b]	406±65[b]	412±70[b]
MTG	158±11	203±16	262±22	326±27	367±25	333±38	320±33[b]	373±42[ab]	383±55[ab]	398±58[ab]	406±64[ab]	411±65[b]	419±68[b]	425±73[b]
NCG	151±10	197±14	252±20	317±25	360±25	324±26	300±35[b]	263±36[b]	321±44[b]	342±48[b]	348±40[b]	371±42[b]	376±45[b]	381±47[b]

a $P < 0.05$ compared with NCG

b $P < 0.05$ compared with VCG

Table S6. Glucose tolerance test (GTT, mmol/L) of all animals. Value=mean ± SD (N=6)

Time (min)	0	15	30	60	90	120	Area under the curve
VCG	4.2±0.43[a]	14.9±1.58[a]	10.7±1.92[a]	6.5±1.17[a]	5.43±0.63[a]	5.22±0.39[a]	1091±117[a]
PCG	9.7±3.79[ab]	22.7±4.22[ab]	21.2±3.9[ab]	19.9±3.55[ab]	18.5±3.51[ab]	16.22±3.7[ab]	2808±311[ab]
MTG	10.1±3.62[ab]	21.8±2.53[ab]	24.1±4.46[b]	21.6±4.83[ab]	20.23±3.97[ab]	16.7±3.01[ab]	3002±467[ab]
NCG	16.8±1.2[b]	26.6±2.12[b]	27.4±0.86[b]	27.2±3.07[b]	26.18±1.71[b]	23.98±1.48[b]	3854±144[b]

a $P < 0.05$ compared with NCG

b $P < 0.05$ compared with VCG

Table S7. Fasting blood glucose levels (mmol/L) of all animals. Value=mean ± SD (N=6)

Time (day)	F	0	3	14	30	45	60
VCG	4.7±0.38	5.18±0.25[a]	5.23±0.66[a]	5.05±0.37[a]	5.4±0.48[a]	4.75±0.5[a]	4.56±0.52[a]
PCG	5.19±0.43	14.5±6.27[b]	17.14±5.89[b]	16.91±5.94[b]	14.63±5.64[ab]	11.24±5.7[ab]	9.94±4.83[ab]
MTG	5.1±0.46	14.44±4.43[b]	15.21±4.47[b]	14.99±5.06[b]	12.74±3.68[ab]	10.83±4.3[ab]	10.08±3.23[ab]
NCG	4.76±0.48	14.48±6.22[b]	21.81±7.18[b]	19.3±5.66[b]	20.4±1.63[b]	19.94±1.38[b]	18.64±2.43[b]

a $P < 0.05$ compared with NCG

b $P < 0.05$ compared with VCG

Table S8. HbA1c proportion (%) of all animals. Value=mean ± SD (N=6)

	VCG	PCG	MTG	NCG
sample 1	1.6	2.1	2.4	2.6
sample 2	1.5	2.6	2.4	2.9
sample 3	1.5	2.2	2.3	3.3
sample 4	1.9	1.7	2.8	3.7
sample 5	1.6	2.1	1.7	2.4
sample 6	1.8	2.3	2.2	2.9
Mean±SD	1.65±0.16[a]	2.17±0.29[ab]	2.3±0.36[ab]	2.97±0.47[b]

a $P < 0.05$ compared with NCG

b $P < 0.05$ compared with VCG

Table S9. Fasting serum insulin (mU/L) of all animals. Value=mean ± SD (N=6)

	VCG	PCG	MTG	NCG
sample 1	5.88	7.1	4.96	7.91
sample 2	6.07	6.66	4.97	5.94

sample 3	5.66	5.81	5.96	6.66
sample 4	6.42	5.74	6.7	6.22
sample 5	6.99	6.19	6.85	10.3
sample 6	8.33	9.34	8.42	8
Mean±SD	6.56±0.98	6.81±1.34	6.31±1.31	7.5±1.6

a P < 0.05 compared with NCG

b P < 0.05 compared with VCG

Table S10. HOMA-IR of all animals. Value=mean ± SD (N=6)

	VCG	PCG	MTG	NCG
sample 1	1.23	4.57	1.98	7.03
sample 2	1.24	3.38	2.48	3.96
sample 3	1.23	2.69	2.38	5.45
sample 4	1.37	2.45	4.05	4.73
sample 5	1.34	2.03	3.1	9.77
sample 6	1.85	3.86	2.88	7.04
Mean±SD	1.38±0.24[a]	3.16±0.95[ab]	2.81±0.72[ab]	6.33±2.09[b]

a P < 0.05 compared with NCG

b P < 0.05 compared with VCG

Table S11. HOMA-IS of all animals. Value=mean ± SD (N=6)

	VCG	PCG	MTG	NCG
sample 1	28.1	11.0	12.4	8.9
sample 2	29.7	13.2	9.99	8.91
sample 3	26.0	12.6	14.9	8.14
sample 4	30.1	13.5	11.1	8.19
sample 5	36.6	18.8	15.1	10.8
sample 6	37.5	22.6	24.6	9.09
Mean±SD	31.3±4.66[a]	15.3±4.46[ab]	14.7±5.27[ab]	9.01±0.96[b]

a P < 0.05 compared with NCG

b P < 0.05 compared with VCG

Table S12. HOMA-β (%) of all animals. Value=mean ± SD (N=6)

	VCG	PCG	MTG	NCG
sample 1	97.9	12.9	18.0	9.59
sample 2	110	16.9	12.9	10.3
sample 3	80.9	16.8	21.7	8.94
sample 4	98.8	18.8	13.3	9.15
sample 5	174	31.7	20.4	11.5
sample 6	111	32.2	40.1	9.81
Mean±SD	112±32.5[a]	21.6±8.29[ab]	21.1±9.99[ab]	9.88±0.92[b]

a P < 0.05 compared with NCG

b P < 0.05 compared with VCG

Table S13. Clinical chemistry parameters of all animals. Value=mean ± SD (N=6)

	TG	CHO	HDL	LDL	ALT	AST	ALP
VCG	0.42±0.1[a]	1.13±0.16[a]	1.18±0.14	0.27±0.03[a]	46.2±7.7[a]	254±32.7[a]	74.5±5.3[a]
PCG	0.45±0.19[a]	1.12±0.2[a]	1.14±0.18	0.21±0.02[a]	49.2±14.8	200±50[a]	164±48[ab]
MTG	0.4±0.26[a]	1.1±0.44[a]	1.08±0.16	0.22±0.07[a]	51.7±18.7	185±40[a]	186±60.2[ab]
NCG	1.06±0.34[b]	1.6±0.33[b]	1±0.15	0.58±0.15[b]	60.3±21.6[b]	130±28.2[b]	320±79.7[b]

a P < 0.05 compared with NCG

b P < 0.05 compared with VCG

Table S14. Serum NEFAs levels of all animals. Value=mean ± SD (N=6)

	VCG	PCG	MTG	NCG
sample 1	0.57	0.33	0.42	0.74
sample 2	0.73	0.35	0.34	0.63
sample 3	0.66	0.56	0.11	0.62
sample 4	0.77	0.38	0.31	1
sample 5	0.43	0.39	0.27	1.02
sample 6	0.59	0.58	0.5	0.65
Mean±SD	0.63±0.12	0.43±0.11[ab]	0.33±0.13[ab]	0.78±0.19

a P < 0.05 compared with NCG

b P < 0.05 compared with VCG

Table S15. Taxa annotated of intestinal microflora. Value=mean ± SD (N=6)

Taxa annotated	VCG3	PCG3	MTG3	NCG3
The total of Proteobacteria	218±91	412±118[ab]	238±93.4[b]	154±95.4
The total of Firmicutes	20099±928	20814±467	16746±1161[a]	18422±4139
The total of Bacteroidetes	1988±988[b]	945±302[a]	3154±1233[b]	696±283[a]
The total of Actinobacteria	375±132	408±136	2584±1823[a]	3515±4239
Verrucomicrobia|Verrucomicrobiae|Verrucomicrobiales|Verrucomicrobiaceae|Akkermansia	5±4.5	32±32	2±2.3	6±8.8
Tenericutes|Mollicutes|RF39	93±41.9[b]	163±132.1[b]	48±19.1[ab]	14±7.1[a]
ProteoGammaproteoPseudomonadales|Pseudomonadaceae|Pseudomonas	77±32.4[b]	76±31.2[b]	51±30.8	30±22.9[a]
ProteoGammaproteoPseudomonadales|Moraxellaceae|Acinetobacter	21±10.8	27±13.3	15±9.2	12±7.7
ProteoGammaproteoOceanospirillales|Halomonadaceae|Halomonas	2±1.2	3±1.9	3±1.8	1±1
ProteoGammaproteoEnterobacteriales|Enterobacteriaceae|Escherichia	13±10	17±8.7	33±44.8	18±10.1
ProteoDeltaproteoDesulfovibrionales|Desulfovibrionaceae	47±24.3	251±170[ab]	48±15.1	69±53.2
ProteoBetaproteoBurkholderiales|Comamonadaceae|Tepidimonas	2±3.1	2±2	1±0.9	1±1.6
ProteoBetaproteoBurkholderiales|Alcaligenaceae|Sutterella	36±25.1[b]	8±3.6[a]	69±38.7[b]	7±5.2[a]
ProteoAlphaproteoSphingomonadales|Sphingomonadaceae|Sphingopyxis	4±1.2	11±5.9[ab]	5±3.4	2±1.7
ProteoAlphaproteoSphingomonadales|Sphingomonadaceae|Sphingomonas	1±1.2	1±1.5	1±1.2	8±15
ProteoAlphaproteoRhizobiales|Phyllobacteriaceae|Aminobacter	10±5.3[b]	9±2.3[b]	6±3.3	5±2.9[a]
ProteoAlphaproteoRhizobiales|Brucellaceae|Ochrobactrum	5±1.5	6±2.4[b]	4±1.7	3±2.4
ProteoAlphaproteoRF32	2±4.4	1±0.8	4±4.7	1±1.6
Firmicutes|Erysipelotrichi|Erysipelotrichales|Erysipelotrichaceae|Holdemania	2±1.5	5±2.2	7±5.4	3±1.3
Firmicutes|Erysipelotrichi|Erysipelotrichales|Erysipelotrichaceae|Eubacterium	26±10.7	545±457[a]	95±61.3	514±574
Firmicutes|Erysipelotrichi|Erysipelotrichales|Erysipelotrichaceae|Coprobacillus	52±77.3[b]	286±262	37±16.1[b]	635±459[a]
Firmicutes|Erysipelotrichi|Erysipelotrichales|Erysipelotrichaceae|Clostridium	7±5.3	21±12.5[ab]	24±19.5	5±4.6
Firmicutes|Erysipelotrichi|Erysipelotrichales|Erysipelotrichaceae|Allobaculum	43±11.1	133±129	97±97.1	501±735
Firmicutes|Erysipelotrichi|Erysipelotrichales|Erysipelotrichaceae	105±43.8	183±72.1[b]	70±34.7[a]	66±55.2

Firmicutes|Clostridia|Clostridiales|Veillonellaceae|Phascolarctobacterium	244±137.6^{b}	56±27^{a}	374±319.8	52±34.5^{a}
Firmicutes|Clostridia|Clostridiales|Ruminococcaceae|Ruminococcus	1279±410^{b}	338±52.8^{a}	769±335	328±186^{a}
Firmicutes|Clostridia|Clostridiales|Ruminococcaceae|Oscillospira	524±169.9^{b}	342±50.1^{a}	715±650.4	262±153.5^{a}
Firmicutes|Clostridia|Clostridiales|Ruminococcaceae|Butyricicoccus	3±0.9^{b}	9±4.4^{ab}	2±1.5	1±1^{a}
Firmicutes|Clostridia|Clostridiales|Ruminococcaceae	4166±626^{b}	3036±599^{ab}	3375±1157	2251±864^{a}
Firmicutes|Clostridia|Clostridiales|Peptococcaceae|rc4-4	8±6.7	19±9^{a}	15±10.5^{b}	7±5.6
Firmicutes|Clostridia|Clostridiales|Mogibacteriaceae	145±27.1	308±95.8^{ab}	151±21.5	117±54.6
Firmicutes|Clostridia|Clostridiales|Lachnospiraceae|Ruminococcus	481±97.7^{b}	239±62.2^{ab}	222±46.5^{ab}	114±43.1^{a}
Firmicutes|Clostridia|Clostridiales|Lachnospiraceae|Roseburia	198±121.7^{b}	45±11.3^{a}	105±48.6	51±27.6^{a}
Firmicutes|Clostridia|Clostridiales|Lachnospiraceae|Dorea	1001±334^{b}	2268±636^{a}	665±167^{ab}	2041±540^{a}
Firmicutes|Clostridia|Clostridiales|Lachnospiraceae|Coprococcus	640±118.2^{b}	268±85.3^{a}	526±157.3	349±153.7^{a}
Firmicutes|Clostridia|Clostridiales|Lachnospiraceae|Clostridium	14±5.4	11±6.4	13±8.7	10±3.8
Firmicutes|Clostridia|Clostridiales|Lachnospiraceae|Blautia|producta	366±93^{b}	1694±239^{a}	455±110^{b}	1152±471^{a}
Firmicutes|Clostridia|Clostridiales|Lachnospiraceae|Blautia	1447±270	2579±664^{a}	1263±231.1	1806±704
Firmicutes|Clostridia|Clostridiales|Lachnospiraceae|Anaerostipes	12±6.6^{b}	3±2.3^{a}	5±1.6^{a}	4±1.7^{a}
Firmicutes|Clostridia|Clostridiales|Lachnospiraceae	2530±169	4555±989^{a}	3062±683	3983±1585
Firmicutes|Clostridia|Clostridiales|Eubacteriaceae|Anaerofustis	14±6.5	10±2.7	6±2.5	6±5.2
Firmicutes|Clostridia|Clostridiales|Dehalobacteriaceae|Dehalobacterium	8±4.6^{b}	3±2.8^{a}	3±2.2	1±0.5^{a}
Firmicutes|Clostridia|Clostridiales|Clostridiaceae|Clostridium	231±74^{b}	34±10.9^{a}	110±71.1^{ab}	34±20.7^{a}
Firmicutes|Clostridia|Clostridiales|Clostridiaceae	11±4.5	17±13.5	12±7	11±3.8
Firmicutes|Clostridia|Clostridiales|Christensenellaceae|Christensenella	13±4.1	10±2.7	10±1.5	9±4.5
Firmicutes|Clostridia|Clostridiales|Christensenellaceae	56±8.8	64±14.4	103±47.6	54±16.6
Firmicutes|Clostridia|Clostridiales	2682±844^{b}	1985±352^{b}	2122±536^{b}	955±456^{a}
Firmicutes|Bacilli|Turicibacterales|Turicibacteraceae|Turicibacter	25±12.6^{b}	91±46.1^{a}	95±73.2^{a}	79±51.1^{a}
Firmicutes|Bacilli|Lactobacillales|Streptococcaceae|Streptococcus	10±2.5^{b}	97±28.2^{ab}	20±10.7	47±34^{a}
Firmicutes|Bacilli|Lactobacillales|Streptococcaceae|Lactococcus	275±214	283±139^{b}	92±87.9	95±92.4

Firmicutes|Bacilli|Lactobacillales|Lactobacillaceae|Lactobacillus	3466±1148	1230±244[a]	2093±1803	2867±1905
Firmicutes|Bacilli|Lactobacillales|Enterococcaceae|Enterococcus	15±5.4	31±24.8	32±31.9	11±3.7
Firmicutes|Bacilli|Lactobacillales|Enterococcaceae	2±1.2	15±11.8[b]	4±4.1	3±2.3
Firmicutes|Bacilli|Lactobacillales|Carnobacteriaceae|Granulicatella	1±1.4	2±2.9	1±1.2	0±0
Firmicutes|Bacilli|Bacillales|Staphylococcaceae|Staphylococcus	1±0.8	3±0.8[ab]	2±0.8[ab]	0±0.4
Elusimicrobia|Elusimicrobia|Elusimicrobiales|Elusimicrobiaceae|Elusimicrobium	3±0.8[b]	1±0.8[a]	3±4.4	0±0[a]
Cyano4C0d-2|YS2	11±8.2[b]	5±3.5	17±17.4	1±2.4[a]
Bacteroidetes|Bacteroidia|Bacteroidales|S24-7	659±241[b]	471±258	1439±841[b]	314±110[a]
Bacteroidetes|Bacteroidia|Bacteroidales|Rikenellaceae	27±18.5[b]	7±5.8[a]	11±7.4	3±1.5[a]
Bacteroidetes|Bacteroidia|Bacteroidales|Prevotellaceae|Prevotella	975±664[b]	123±27[a]	1005±505[b]	87±48.1[a]
Bacteroidetes|Bacteroidia|Bacteroidales|Porphyromonadaceae|Parabacteroides	38±37.3	56±10.4	128±80.6[ab]	47±31.1
Bacteroidetes|Bacteroidia|Bacteroidales|Paraprevotellaceae|Prevotella	55±18.9[b]	31±14.3[b]	35±25.6[b]	6±3.7[a]
Bacteroidetes|Bacteroidia|Bacteroidales|Paraprevotellaceae|Paraprevotella	1±2	0±0.8	3±6.9	1±0.5
Bacteroidetes|Bacteroidia|Bacteroidales|Odoribacteraceae|Odoribacter	9±6.9[b]	1±0.8[a]	2±3.2	0±0a
Bacteroidetes|Bacteroidia|Bacteroidales|Odoribacteraceae|Butyricimonas	6±5.1	6±2.3	28±26.2	4±2.4
Bacteroidetes|Bacteroidia|Bacteroidales|Bacteroidaceae|Bacteroides	190±175	248±64.2	500±212[a]	230±133
Bacteroidetes|Bacteroidia|Bacteroidales	28±27.3	4±2.7	5±3.6	5±12.2
ActinoCoriobacteriia|Coriobacteriales|Coriobacteriaceae|Collinsella	35±14	130±70.9[a]	29±11.6	199±212
ActinoCoriobacteriia|Coriobacteriales|Coriobacteriaceae|Adlercreutzia	135±61.2[b]	58±14[a]	89±29.9	63±28[a]
ActinoCoriobacteriia|Coriobacteriales|Coriobacteriaceae	150±59.8	68±16.2[a]	327±96.3[a]	311±305
ActinoActinoBifidobacteriales|Bifidobacteriaceae|Bifidobacterium	37±30.4[b]	117±63.6[ab]	2124±1726[a]	2924±4347[a]
ActinoActinoBifidobacteriales|Bifidobacteriaceae	0±0.4[b]	3±1.2[a]	0±0.5[b]	2±1.4[a]
ActinoActinoActinomycetales|Nocardioidaceae|Aeromicrobium	4±1.6[b]	3±1.9	2±1.4	2±1.5[a]
ActinoActinoActinomycetales|Nocardiaceae|Rhodococcus	2±1.8	1±0.5	2±0.5	1±0.8
ActinoActinoActinomycetales|Micrococcaceae|Rothia	12±3.7	24±18.7	9±4.9[a]	11±5.9
ActinoActinoActinomycetales|Brevibacteriaceae|Brevibacterium	2±1.5	4±2.3	2±1.3	2±2

a, $P < 0.05$ compared with VCG
b, $P < 0.05$ compared with NCG
